# Explainable fine-grained feature enhancement framework for strawberry diagnostic decision making

**DOI:** 10.3389/fpls.2026.1941698

**Published:** 2026-10-09

**Authors:** Hye-Young Wang, Seoung-Ho Choi

**Affiliations:** 1 Department of AI Application, Hansung University, Seoul, Republic of Korea; 2 Department of Biomedical Informatics, Korea University College of Medicine, Seoul, Republic of Korea; 3 Faculty of Basic Liberal Art, Hansung University, Seoul, Republic of Korea

**Keywords:** data leakage, explainable AI (XAI), fine-grained feature enhancement, grouped cross-validation, smart agriculture, strawberry disease diagnosis

## Abstract

As the number of smart agricultural automation systems increases, accurate disease diagnosis is essential to maximize production efficiency. However, early-stage symptoms of strawberry diseases (e.g., Tipburn, Phytophthora blight, and Fusarium wilt) often manifest as low-contrast micro-symptoms that are visually similar to the inherent complex texture of strawberry leaves, leading to high technical difficulties in achieving an accurate diagnosis. Furthermore, providing an explanation for the diagnosis remains challenging, which hinders trust in field applications. We present EN-FDEA, a deep learning framework that integrates an EfficientNetV2 backbone with a Convolutional Block Attention Module (CBAM) and a Fine-grained Discriminative Enhancement (FDEA) module combining depthwise separable convolution, parallel feature amplification, and channel recalibration, and we evaluate it together with a provenance audit of the corpus on which it is trained. Parsing the acquisition metadata embedded in every file name showed that the 1,000 abnormal images of each disease originate from only 33–73 individual plants, and that normal and abnormal images share no greenhouse; under the image-level cross-validation used in the original design, 91.9–99.8% of abnormal plants appear in both the training and the test partition of a fold. All results reported here are therefore obtained under plant-disjoint cross-validation with five random seeds, with a site-disjoint arm as the strictest comparison. Removing plant leakage lowers balanced accuracy by up to 0.082 and removing site overlap by up to 0.173. Under the leakage-free protocol, EN-FDEA performs comparably to, and not reliably better than, its EfficientNetV2 backbone (mean paired balanced-accuracy differences of –0.013 to +0.012 across datasets and conditions); the only Holm-corrected significant advantage is Phytophthora blight under overexposure (+0.012, p = 0.047). Forensic analysis further shows that the elevated performance previously observed under synthetic overexposure is a property of the transform: a logistic-regression classifier using only global exposure statistics reaches balanced accuracies of 0.81–0.92, and models trained on original images transfer poorly to synthetic overexposure while transferring well to genuinely over-illuminated frames selected from the corpus itself. We report explanation faithfulness, calibration, and measured deployment cost, and we state where the corpus structure, rather than the model, bounds what can be concluded. Code, split definitions, and per-image predictions are released so that every number can be regenerated.

## Graphical Abstract

**Figure:**
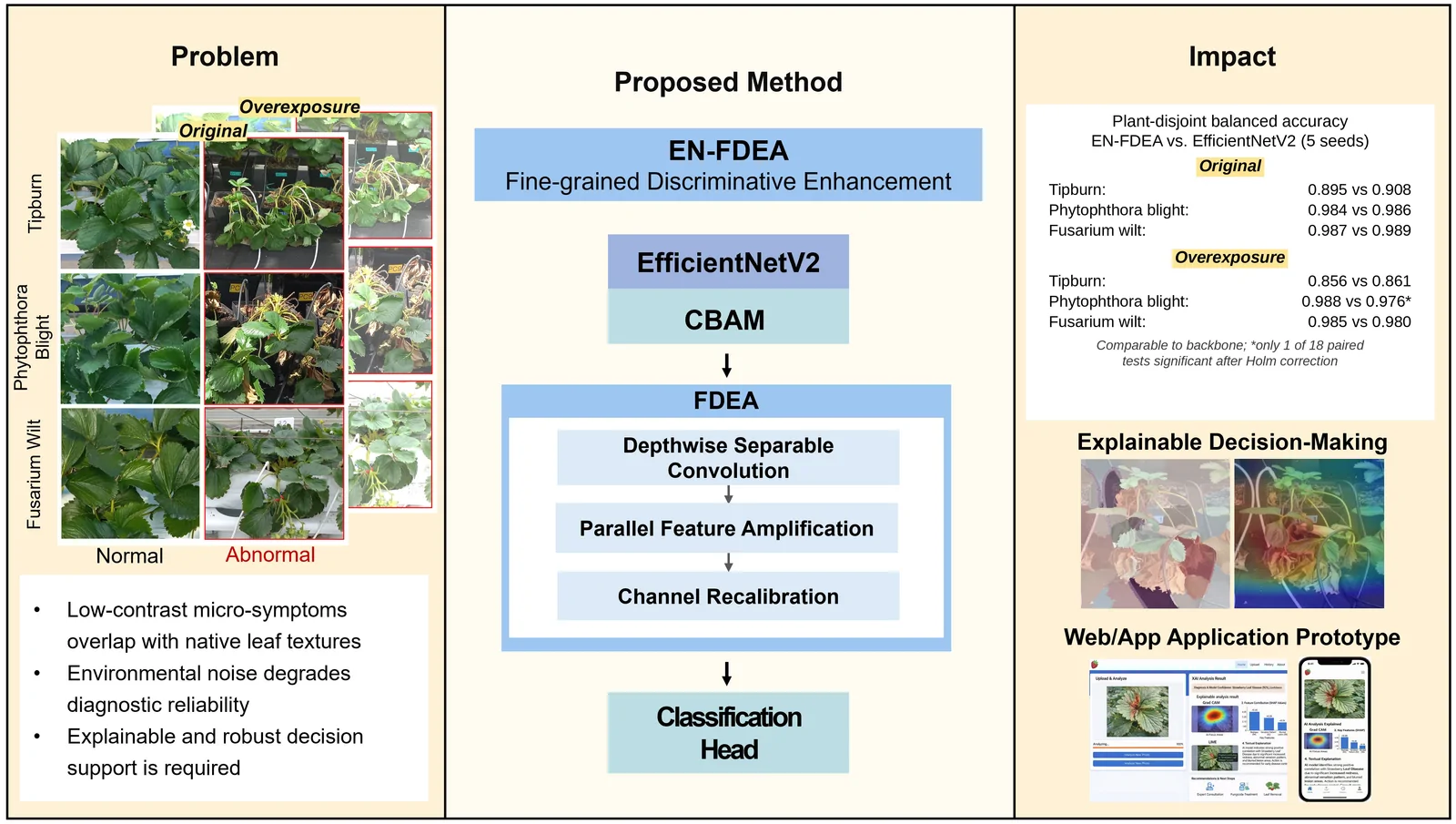

## Highlights

- Plant and greenhouse identifiers recovered from file names expose leakage in image-level splits.
- Normal and abnormal images share no greenhouse, confounding disease label with acquisition site.
- Under plant-disjoint evaluation with five seeds, EN-FDEA matches its EfficientNetV2 backbone.
- Exposure-only classifiers reach 0.81–0.92 balanced accuracy, bounding the illumination shortcut.
- Deployment cost, calibration, and explanation faithfulness are reported with released code and splits.

## 1 Introduction

Smart agricultural automation systems are key technologies for alleviating the problem of agricultural labor shortages and maximizing production efficiency (Kamilaris and Prenafeta-Boldú, 2018; Liakos et al., 2018). For such systems to be effective in actual cultivation sites, high-performance computer vision-based diagnostic technology that can accurately determine the growth status of crops and whether they are diseased or have physiological disorders must be in place (Kamilaris and Prenafeta-Boldú, 2018).

In particular, strawberries, a representative high-income crop in facility gardening, are sensitive to environmental changes and vulnerable to various diseases, which can directly lead to serious yield reductions if not detected early. Therefore, automated and quality control methods are essential for capturing minute changes in leaves, which are key indicators of health status throughout strawberry growth.

In strawberry cultivation environments, early symptoms are mainly expressed on leaves, with Tipburn, Phytophthora blight, and Fusarium wilt, starting with microscopic spots, discoloration, or local tissue changes on the leaf surface. However, because strawberry leaves contain complex lobe structures and natural color changes, and morphological diversity exists depending on the growth stage, visually distinguishing normal tissues from abnormal lesions in the early stages is difficult (Barbedo, 2016).

This biological ambiguity, combined with external factors arising from the image acquisition process, threatens the reliability of diagnostic systems. High-quality image data must be secured for precise pathological analysis; however, this is not easy because of the uncontrollable nature of agricultural fields. Moreover, in smart farms and on-site forecasting environments, various environmental disturbances such as lighting changes, photographing angles, blur caused by leaf shaking, sensor noise, and image quality deterioration due to dust and moisture occur simultaneously. This realistic noise makes the micro-parallelization of the leaf surface unclear, which is a major factor that hinders the robustness performance of existing automatic diagnosis models (Barbedo, 2016; Barbedo, 2018; KC et al., 2021). Therefore, there is an urgent need for a technical approach to maintain stable diagnostic performance and ensure the reliability of judgments, even under real-world field conditions (Liakos et al., 2018).

In response to these needs, deep learning technology, which has excellent performance in feature extraction and pattern recognition of unstructured data, has emerged as a key alternative for solving challenges in agriculture. Recently, disease diagnosis research using convolutional neural networks (CNNs) and transformer-based models has been actively conducted; however (Mohanty et al., 2016; Borhani et al., 2022), a number of prior studies are limited to high-quality images or cases with distinct morbidity collected in controlled environments (Barbedo, 2016; Barbedo, 2018). Consequently, limitations of rapidly deteriorating diagnostic performance have been reported for the initial morbidity of low contrast that occurs in actual cultivation environments (Barbedo, 2016; Barbedo, 2018).

In addition, deep learning-based diagnostic models have the characteristic of being “black-boxes”, where the decision-making process is internally opaque despite their high classification performance (Mostafa et al., 2023), which acts as an entry barrier for field workers to trust and utilize the model’s judgment, especially in agricultural environments where rapid early response is required, and explainability to understand the basis of diagnostic results is essential (Lundberg and Lee, 2017; Selvaraju et al., 2017; Wei et al., 2022).

To overcome these limitations, we aim to simultaneously secure robust diagnostic performance and explainability, despite uncertainties in actual growing environments. In the course of this revision, however, an audit of the corpus on which such claims are normally evaluated changed what this paper is able to conclude, and the contributions below are stated in the corrected form. Section 3.2 reports the audit; Sections 4.3–4.11 report the results obtained under the corrected protocol.

The contributions of this revised study are as follows.

- We audit the provenance of a nationally released strawberry disease corpus at the level of individual plants, greenhouses and acquisition dates, and we quantify how much of the previously reported performance depends on evaluation protocol rather than on pathology.
- We report that disease label and acquisition site are completely confounded in this corpus, and we bound the resulting shortcut with site-disjoint evaluation, cue-only classifiers and destructive probes.
- We propose EN-FDEA, an EfficientNetV2-based diagnostic model incorporating the Fine-grained Discriminative Enhancement (FDEA) module, and we evaluate it under plant-disjoint cross-validation with five random seeds against scratch-trained and ImageNet-pretrained baselines, with paired significance testing and multiplicity correction.
- We show that the elevated performance previously reported under synthetic overexposure is attributable to the transform rather than to robustness, using exposure statistics, cue-only probes, destructive probes, cross-condition transfer and a held-out set of genuinely over-illuminated frames.
- We characterise the framework as a decision-support prototype rather than a validated field deployment, reporting explanation faithfulness, calibration and measured parameter, computation and latency cost, and we release code, split definitions and per-image predictions.

## 2 Related works

### 2.1 Deep learning in agricultural and crop analysis

With recent developments in precision agriculture, deep-learning-based image analysis techniques have been actively studied for the automatic diagnosis of crop diseases (Mohanty et al., 2016; Kamilaris and Prenafeta-Boldú, 2018). Crop diseases are the main cause of production loss and quality degradation, and early detection and accurate diagnosis play key roles in improving agricultural productivity. However, conventional visual inspection or expert-based diagnosis is time-consuming and expensive, and has limitations that rely on subjective judgment. To solve this problem, a deep learning model centered on convolutional neural networks (CNNs) was introduced for crop disease classification. By automatically learning the visual features of the disease from large-scale image data, it demonstrated superior performance over conventional methods (Ferentinos, 2018). Nevertheless, crop images obtained in actual cultivation environments have high variability in disease patterns owing to lighting changes, complex backgrounds, and differences in appearance depending on the growth stage, which hinders model performance (Barbedo, 2018). Accurately capturing low-contrast disease signals, such as fine color changes or local tissue damage, remains an important challenge, particularly in field-image-based disease diagnosis.

### 2.2 Feature enhancement technique for fine pathological features

The symptoms in crop disease images are usually localized and often have textures similar to those of normal growing tissues. Owing to these characteristics, the standard CNN structure is at risk of overreacting to background information or irrelevant patterns rather than the core features corresponding to the disease (Barbedo, 2016). To compensate for this, recent studies have proposed a feature enhancement module that selectively emphasizes features that are important for classification. Representative methods include squeeze-and-excitation (SE) networks based on channel attention, which dynamically adjust channel-specific importance to highlight meaningful features (Hu et al., 2018). CBAM has since been extended to simultaneously learn which features and where to focus by combining channel and spatial attention (Woo et al., 2018). It has been reported that these attention-based techniques are effective in amplifying microscopic color changes or tissue damage caused by disease. Multiscale feature extraction has also been studied as an important approach because the sizes and forms of diseases vary. The inception module or atrous convolution simultaneously captures disease patterns of various sizes by utilizing different accommodation areas in parallel (Szegedy et al., 2015; Chen et al., 2018). However, crop disease images acquired in an actual cultivation environment often appear as a mixture of low-contrast micro-features and complex background noise, and it has been reported that simple multi-scale feature extraction alone has limitations in effectively separating the disease signal and background information (Barbedo, 2016; KC et al., 2021).

### 2.3 Decision making and explainability in agricultural artificial intelligence

For practical use of AI-based diagnostic systems in agriculture, the reliability of the model’s decision-making process and high classification performance are essential. An incorrect disease diagnosis can lead to unnecessary pesticide use or crop disposal, causing economic losses and environmental problems (Liakos et al., 2018). Therefore, there is a continuously increasing demand for explainable AI (XAI) that can explain the basis on which the model judges the disease. Grad-CAM is a representative technique that intuitively presents the basis for the judgment of a model by visually representing the areas that contribute to the prediction results of the CNN. This technique is used as a tool to verify whether the model focuses on the actual lesion area in crop disease classification and plant stress analysis (Kuo et al., 2023; Murugesan et al., 2025). In addition, LIME and SHAP can quantify the contribution of input features independently of the model structure, which is utilized to interpret predictive grounds and evaluate model reliability (Ribeiro et al., 2016; Lundberg and Lee, 2017), including in agricultural image-based disease diagnosis (Nasir et al., 2025; Pal et al., 2025; Shafik et al., 2025).

### 2.4 Research gaps and limitations

Existing deep learning studies have demonstrated remarkable results in crop disease diagnosis; however, there is a limit to the complexity of actual strawberry cultivation sites. First, existing models cannot clearly distinguish the initial micro-morbidities of low contrast in an environment where dense foliage and natural discoloration unique to strawberry leaves are mixed. Second, the diagnostic performance is unstable because of the lack of robustness against abnormal field noise, such as overexposure. Third, it is difficult to guarantee field reliability because of the lack of explainability that visually presents the basis for judgment beyond simple classification. Therefore, we differ from previous studies by proposing an integrated diagnosis framework that simultaneously diagnoses the precision capture of micro-features, robustness against environmental disturbance, and explainability. A fourth gap became apparent during this revision and is now central to the paper. Work published since our original submission reports strong results for strawberry leaf disease detection under conventional evaluation (He et al., 2024), while a survey of plant-disease corpora identifies acquisition structure — repeated images of the same plant, class-specific collection sites, and the resulting leakage — as a systematic source of optimistic performance in exactly this literature (Xu et al., 2024). The same failure modes are documented outside agriculture, in applied machine learning generally (Kaufman et al., 2012; Roberts et al., 2021; Varoquaux and Cheplygina, 2022) and in medical imaging, where a model can learn the signature of the site rather than the pathology (Zech et al., 2018). Section 3.2 reports that both conditions hold in the corpus used here, and the rest of this paper is written accordingly.

## 3 Methodology

### 3.1 Problem definition

The key challenges are to simultaneously overcome the difficulty in distinguishing micro-symptoms owing to the complex texture of the surface of strawberry leaves, the problem of deteriorating diagnostic reliability in an automated smart farm forecasting environment, and the lack of explainability. Specifically, we define the problem in the following three technical dimensions by reflecting the limitations of an actual facility horticultural cultivation site.

#### 3.1.1 Distinguishing limitations of micro-symptoms and strawberry leaf native textures

Existing common computer vision models show strength in capturing distinct abnormal features with sufficient morbidity, but have limitations in stably distinguishing between the dense follicle structure of strawberry leaves, natural color changes, and early signs visually similar to the surface texture. Tipburn, Fusarium wilt, and Phytophthora blight often begin with low-contrast features, such as microscopic spots, local discoloration, and tissue changes on the leaf surface, which are mixed with the natural variability of normal tissues, causing feature ambiguity. Consequently, general feature extractors may have difficulty effectively separating the morbidity feature from the background texture, limiting their sensitivity to micro-features.

#### 3.1.2 Deteriorated diagnostic reliability due to environmental noise

In actual smart farm cultivation and field forecasting environments, various confounding factors occur simultaneously, such as time zone and illuminance changes, discrepancy between shooting angle and distance, blur due to leaf shaking, sensor noise, condensation, and dust contamination due to moisture, etc. These noises weaken the abnormal signal by blurring the boundaries of the staging area or distorting the fine pattern on the leaf surface; in contrast, they degrade the diagnostic stability by mistaking the unrelated pattern as staging. Therefore, a robust diagnostic framework is required, the performance of which does not fluctuate rapidly, even under adverse conditions.

#### 3.1.3 Explainability of judgment evidence

High classification performance alone makes it difficult for field workers to trust the diagnosis results and respond immediately. Therefore, beyond simple accuracy, there is a need for an explainable diagnostic system that clearly suggests whether the model was judged to be abnormal based on the visual cues of the leaves, such as follicular adjacent discoloration, edge tissue changes, and morphological patterns of local spots.

### 3.2 Dataset and data preprocessing

#### 3.2.1 Dataset configuration

The dataset consists of images of three representative strawberry disease types, namely Tipburn, Phytophthora blight, and Fusarium wilt. For each disease type, 2,000 images were collected, comprising 1,000 normal and 1,000 abnormal samples, for a total of 6,000 images. The task was defined as a binary classification problem, in which abnormal and normal samples were labeled as 1 and 0, respectively. All images were collected from the Seolhyang cultivar between July and December 2022, spanning the period from planting to harvest under real cultivation conditions in smart-farm environments, including glass and plastic greenhouses at the Gyeongsangnam-do Agricultural Research and Extension Services and commercial farms. To reflect field-realistic acquisition conditions, image collection combined hourly automated capture using fixed mid-range RGB cameras with a resolution of at least 1920×1080 and weekly close-range smartphone photography performed by experts and farm owners. In addition to the original dataset, an overexposure dataset was separately constructed to simulate real cultivation conditions in which bright image regions become saturated due to direct sunlight, reflected light, or strong illumination during image acquisition. Representative examples of the original and overexposure datasets for each disease type are presented in **Figure 1B**.

**Figure 1:**
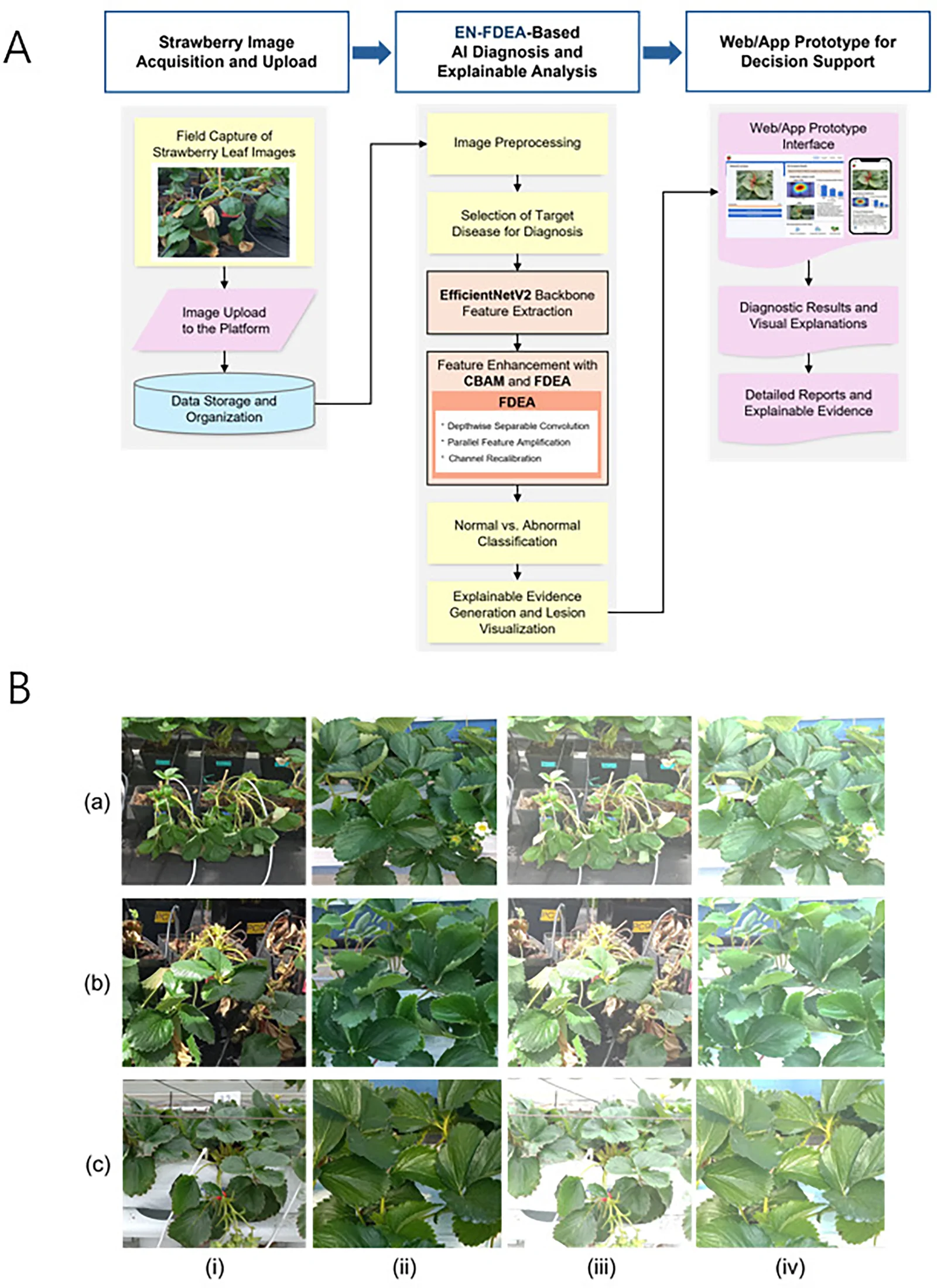
Overview of Strawberry Surface Defect Diagnostic Framework and Dataset Visualization, **(A)** Overall framework of the proposed EN-FDEA-based decision support system for strawberry disease diagnosis, **(B)** Representative samples from each disease-specific dataset. **(a)** Fusarium Wilt, **(b)** Phytophthora Blight, **(c)** Tipburn, **(i)** Original Abnormal, **(ii)** Original Normal, **(iii)** Overexposure Abnormal, and **(iv)** Overexposure Normal.

#### 3.2.2 Acquisition provenance and corpus structure

Every image file in the corpus carries structured acquisition metadata in its name, of the form strawberry_Seolhyang_⟨condition⟩_⟨farm ID⟩_⟨plant ID⟩_⟨YYMMDD⟩⟨HH⟩⟨slot ID⟩.jpg. The submitted version of this work did not use these fields. Parsing them for all 6,000 images establishes two structural properties of the corpus that determine how it can legitimately be evaluated, and both are reported here before any performance result.

First, the abnormal images of each disease originate from very few individual plants. As summarised in **Table 1**, the 1,000 abnormal images comprise 33 plants for Tipburn, 38 for Phytophthora blight and 73 for Fusarium wilt, corresponding to medians of 34, 28 and 11 images per plant, because each plant was photographed hourly over several months. The normal images are drawn from 132–134 plants with a median of 7 images each. Under image-level *k*-fold cross-validation, the measured proportion of abnormal plants that appear in both the training and the test partition of a fold is 99.8%, 99.4% and 91.9% respectively. Different images of the same plant, taken minutes or hours apart, are therefore present on both sides of the split. This is the grouped-leakage failure mode described by Kaufman et al. (2012) and repeatedly identified as a cause of optimistic reporting in applied machine learning (Roberts et al., 2021; Varoquaux and Cheplygina, 2022); Xu et al. (2024) document the same problem specifically for plant-disease image corpora.

**Table 1:** Provenance inventory of the corpus, recovered from acquisition metadata in the file names.

Dataset	Class	Images	Plants	Greenhouses	Images per plant	Plants in both partitions
Tipburn	Normal	1,000	132	37	7	85.3%
	Abnormal	1,000	33	13	34	99.8%
Phytophthora blight	Normal	1,000	134	37	7	83.0%
	Abnormal	1,000	38	12	28	99.4%
Fusarium wilt	Normal	1,000	132	37	7	84.4%
	Abnormal	1,000	73	22	11	91.9%

Images per plant is the median. Plants in both partitions is the measured fraction of that class’s plants appearing in the training and the test partition of the same fold under image-level 3-fold cross-validation, averaged over folds. No greenhouse contributes both classes in any dataset.

Second, disease label and acquisition site are completely confounded. Normal images span 37 greenhouses in every dataset, whereas abnormal images span 13 (Tipburn), 12 (Phytophthora blight) and 22 (Fusarium wilt) greenhouses, with no greenhouse contributing both classes in any of the three datasets. A model can therefore attain a high score by recognising the illumination, background and camera signature of a greenhouse rather than the pathology of a leaf — the agricultural analogue of the site-signature shortcut demonstrated for medical imaging by Zech et al. (2018). This is a property of the corpus as collected, not of any model trained on it; we quantify its contribution in Section 4.9 and treat it as a limitation on what the corpus can support (Section 5).

The corpus is restricted to the Seolhyang cultivar and to Korean greenhouse conditions during a single 2022 growing season. No second cultivar was available within the revision period, and we therefore make no claim of cultivar-general applicability; cross-cultivar validation is listed among the future work in Section 6.

#### 3.2.3 Data preprocessing

To preserve the inherent visual characteristics of images acquired in real cultivation environments and to evaluate robustness under field conditions, preprocessing was intentionally kept to a minimum. All images were resized to 256×256 to ensure input size consistency, and pixel values were normalized to the range of [0,1] for stable optimization. For the overexposure dataset, brightness and contrast were increased by 0.30 and 0.10, respectively, for each original image, after which pixel values were clipped to the range of [0,255]. Subsequently, pixels with values greater than or equal to 235 were forcibly set to 255 to enhance highlight saturation. This setting was designed to evaluate whether the diagnostic model within the proposed framework can directly learn discriminative features from raw images acquired in real cultivation environments. For fair comparison, the same preprocessing procedure was consistently applied to all baseline and comparative models.

The synthetic overexposure condition is a deterministic transform and not a sample of field illumination, and we now report what it does to the images. Averaged over each dataset, it raises the mean pixel value from 106–123 to 183–200, raises the fraction of fully saturated pixels from 0.6–1.8% to 17–26%, and lowers image entropy from 7.3–7.5 to 5.9–6.4 bits. Because the two classes were acquired in different greenhouses and therefore differ in baseline luminance, a fixed clipping threshold does not act identically on them; Section 4.9 measures the resulting class-dependent effect and revises the interpretation of the overexposure experiments accordingly. To evaluate robustness against illumination that was not synthesised, we additionally identified genuinely over-illuminated frames within the corpus itself, without applying any transform, by intersecting the direct-sunlight acquisition hours recorded in the file name (11:00–14:00) with the top decile of measured saturated-pixel fraction. This yields a held-out real-overexposure set of 70 (Tipburn), 62 (Phytophthora blight) and 72 (Fusarium wilt) images, balanced across classes and spanning 41, 27 and 47 plants respectively.

### 3.3 Proposed method

#### 3.3.1 Framework overview

**Figure 1A** illustrates the comprehensive operational framework of the proposed decision support system for strawberry leaf disease diagnosis. The pipeline is systematically divided into three interconnected phases:

1. Strawberry Image Acquisition and Data Management: The process begins with the in-field capture of strawberry leaf images. These raw images are uploaded to a cloud-based platform, where they are systematically organized and stored in a central database to build a robust dataset for model training and evaluation.
2. EN-FDEA-Based AI Diagnosis and Explainable Analysis: This phase serves as the core analytical engine of the framework. Following initial image preprocessing and the selection of target diseases, the system employs an EfficientNetV2 backbone for robust initial feature extraction. To capture subtle and complex disease patterns, the extracted features are refined using a Convolutional Block Attention Module (CBAM) integrated with the proposed FDEA module. The FDEA module specifically enhances feature representation through depthwise separable convolution, parallel feature amplification, and channel recalibration. Subsequently, the enhanced feature maps are utilized to classify the input as normal or abnormal. To ensure model transparency and build trust with end-users, the model generates explainable evidence and visualizes the specific lesion areas using Explainable AI (XAI) techniques.
3. Web and App Prototype for Decision Support and Continuous Learning: In the final deployment phase, the diagnostic results and explainable visual evidence are delivered to end-users (e.g., farmers and agricultural experts) via a user-friendly Web and App prototype interface.

#### 3.3.2 Architecture and functional role of the FDEA framework with EfficientNetV2

**Figure 2** presents the overall architecture of the proposed EN-FDEA model, which integrates the fine-grained discriminative enhancement (FDEA) framework with the EfficientNetV2 backbone. The FDEA framework is designed as a feature enhancement module to further represent low-contrast initial lesion cues and locally distributed fine-grained lesion patterns.

**Figure 2:**
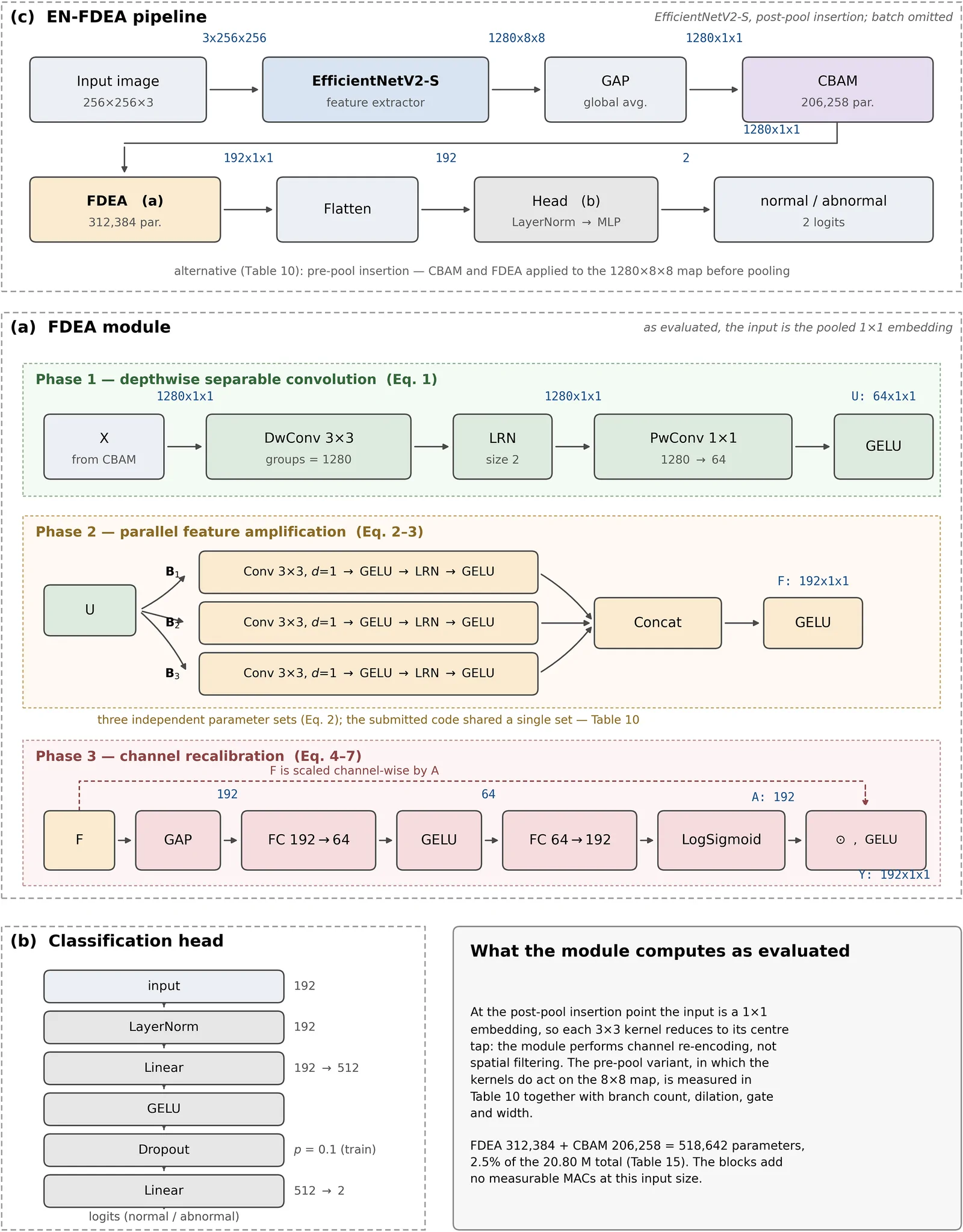
Overall structure of the proposed EN-FDEA model. **(a)** Architecture of the FDEA module, **(b)** architecture of the classification head, and **(c)** complete pipeline of EfficientNetV2 integrated with the FDEA framework. Redrawn for this revision with the tensor shape at every stage for a 256×256×3 input. The insertion point is now stated explicitly: as evaluated, CBAM and the FDEA module are applied after global average pooling, to a 1×1 embedding, so each 3×3 kernel reduces to its centre tap; the pre-pool variant, in which the kernels act on the 8×8 map, is measured in **Table 10**. The three branches of Phase 2 carry independent parameters as specified by Equation 2; the submitted implementation shared a single set. The FDEA and CBAM blocks together account for 518,642 parameters, 2.5% of the 20.80 M total (**Table 15**).

**Figure 2a** illustrates the internal structure of the FDEA module. Given an input feature map, spatial features are extracted using depthwise separable convolution (DSC). DepthwiseConv2D performs independent spatial filtering for each channel, and local response normalization (LRN) is applied to normalize local activation distributions. Subsequently, PointWiseConv2D combines inter-channel information, and the GELU activation is used to introduce a nonlinear feature transformation.

In the next stage, parallel feature amplification is applied. In this stage, Conv2D ( d=1) operations with the same configuration are applied to three parallel paths. Each path consists of Conv2D ( d=1), GELU activation, local response normalization, and another GELU activation. The outputs of the three paths are concatenated along the channel dimension and integrated into a single feature map, which is then passed to the next stage through GELU activation. This process expands the channel dimension and forms an extended feature representation for the subsequent channel recalibration step.

The integrated feature map is then processed by the channel recalibration step. After global average pooling generates a summary vector for each channel, two fully connected layers together with GELU and log-sigmoid activations are sequentially applied to learn channel-wise importance. The resulting channel weights are applied to the feature map through channel-wise multiplication, and the feature responses are recalibrated according to the estimated importance. The recalibrated features are then passed through GELU activation to produce the output of the FDEA module.

**Figure 2b** illustrates the structure of the classification head. The classification head applies LayerNorm to normalize the input feature distribution, followed by a first linear layer for dimension transformation. The GELU activation is then used to introduce nonlinearity, and dropout is applied for regularization during training. Finally, a second linear layer outputs the final binary classification result. This stepwise structure is designed to map the enhanced feature representation from the FDEA module into the decision space.

**Figure 2c** shows the overall structure in which the FDEA framework is inserted after the EfficientNetV2 backbone and the CBAM block. The hierarchical features extracted by EfficientNetV2 are first refined through the CBAM block from channel and spatial perspectives; thereafter, the FDEA module is applied to further reconstruct the feature representation for subsequent classification. Finally, the feature map is converted into a fixed-length vector through global average pooling and flattening and is then fed into the classification head to predict normal or abnormal pathological conditions.

In summary, the FDEA framework is a feature enhancement mechanism that combines spatial feature extraction based on depthwise separable convolution, channel dimension expansion through the Parallel Feature Amplification step, and channel recalibration. By integrating this framework with the EfficientNetV2 backbone, the model is designed to form feature representations for low-contrast and spatially localized lesion patterns.

#### 3.3.3 Algorithm overview of the FDEA module

(**Algorithm 1** presents the overall operational procedure of the proposed Fine-grained Discriminative Enhancement (FDEA) module in pseudocode form. The FDEA module consists of three stages. The first stage performs spatial feature extraction based on depthwise separable convolution. The second stage is the Parallel Feature Amplification stage, which employs three independent parallel Conv2D paths with the same hyperparameter configuration. The third stage is the Channel Recalibration stage, which learns channel-wise importance from the fused feature representation. This stepwise structure was designed as a feature enhancement mechanism to capture low-contrast early symptoms and locally distributed fine-grained lesion patterns.

ALGORITHM 1

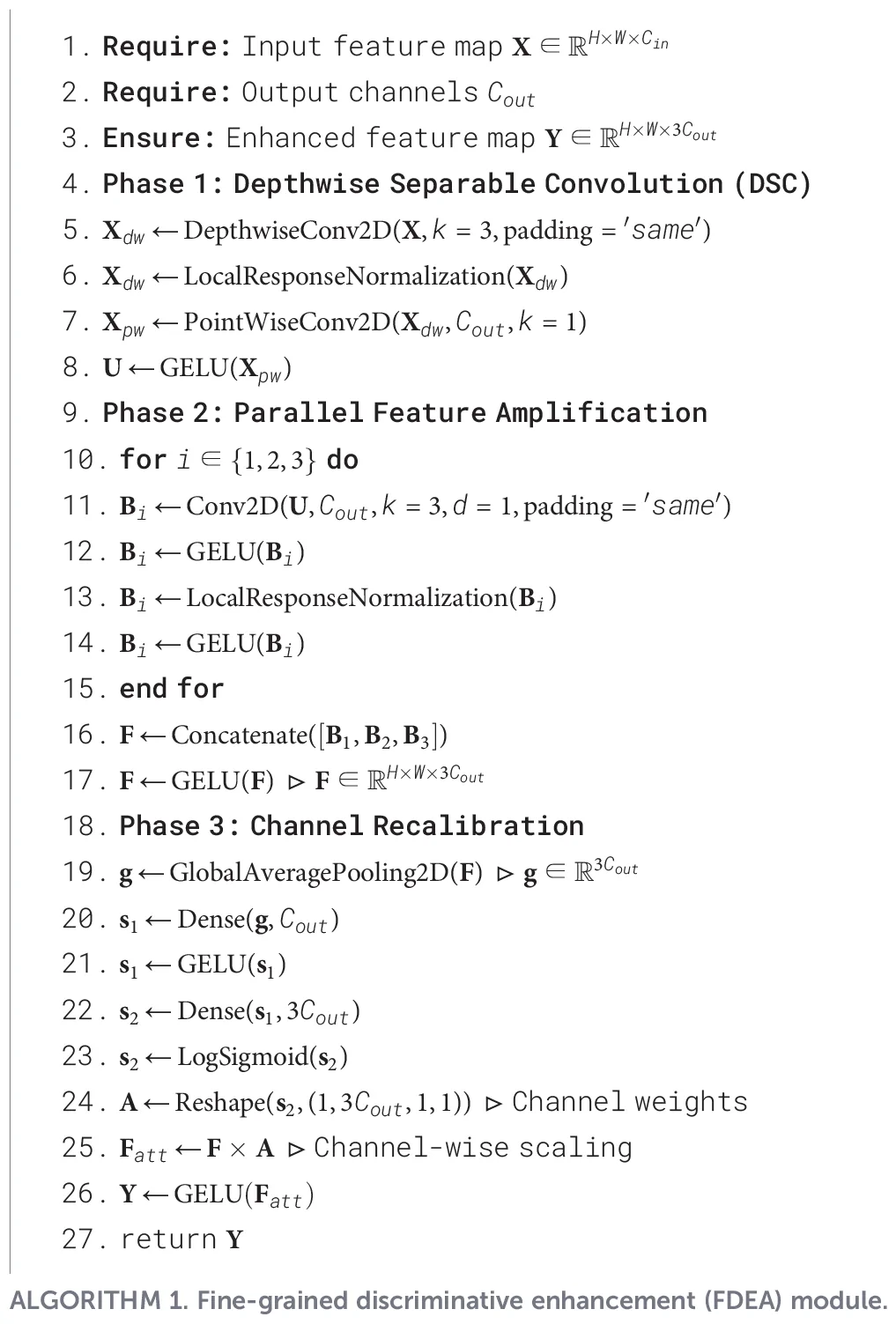

In Phase 1, depthwise separable convolution is applied to extract spatial features from the input feature map $X\in {\mathbb{R}}^{H\times W\times C_{\text{in}}}$, thereby maintaining computational efficiency. First, ${\text{DepthwiseConv}}_{3\times 3}$ performs channel-wise spatial filtering, followed by local response normalization (LRN) to normalize local activation distributions. Subsequently, ${\text{Conv}}_{1\times 1}$ integrates inter-channel information, and a GELU activation is applied to form nonlinear feature responses. This process is defined as follows:

(1) $$U=\text{GELU}({\text{Conv}}_{1\times 1}(\text{LRN}({\text{DepthwiseConv}}_{3\times 3}(X))))$$

In **Equation 1**, **U** denotes the intermediate feature map after depthwise separable convolution and is represented as $U\in {\mathbb{R}}^{H\times W\times C_{\text{out}}}$.

Phase 2 performs the parallel feature amplification step. In this stage, three parallel Conv2D paths with the same structure and configuration are used, each with its own independent parameters. Each parallel path consists of ${\text{Conv}}_{3\times 3}^{\left(d=1\right)}$, GELU activation, LRN, and another GELU activation. The output of each path is defined as follows:

(2) $$B_{i}=\text{GELU}(\text{LRN}(\text{GELU}({\text{Conv}}_{3\times 3}^{\left(d=1\right)}(U)))),\;i\in \{1,2,3\}$$

In **Equation 2**, the output of each parallel path, $B_{i}$, is represented as $B_{i}\in {\mathbb{R}}^{H\times W\times C_{\text{out}}}$. The outputs of the three paths are then concatenated along the channel axis and passed through GELU activation to generate the fused feature map:

(3) $$F=\text{GELU}(\text{Concat}[B_{1},B_{2},B_{3}])$$

In Equation 3, **F** denotes the fused feature map obtained by concatenating the outputs of the three parallel paths along the channel axis, and it is defined as $F\in {\mathbb{R}}^{H\times W\times 3C_{\text{out}}}$.

In Phase3, channel recalibration is performed to learn the channel-wise importance from the fused feature representation. First, global average pooling (GAP) is applied to aggregate spatial information into a channel descriptor.

(4) g=GAP(F)

In Equation 4, **g** denotes the global channel descriptor and is represented as $g\in {\mathbb{R}}^{3C_{\text{out}}}$.

Subsequently, the inter-channel dependencies are modeled using two fully connected layers. The GELU activation is applied after the first linear transformation, and the log sigmoid activation is applied after the second linear transformation to learn the channel weights.

(5) $$s_{1}=\text{GELU}(W_{1}g),\;s_{2}=\text{LogSigmoid}(W_{2}s_{1})$$

In Equation 5, $s_{1}\in {\mathbb{R}}^{C_{\text{out}}}$ and $s_{2}\in {\mathbb{R}}^{3C_{\text{out}}}$. In addition, the learnable weight matrices are defined as $W_{1}\in {\mathbb{R}}^{C_{\text{out}}\times 3C_{\text{out}}}$ and $W_{2}\in {\mathbb{R}}^{3C_{\text{out}}\times C_{\text{out}}}$.

The learned channel weights are reshaped and applied to the fused feature map through channel-wise multiplication. Finally, GELU activation is applied to generate the final output of the FDEA module:

(6) $$A=\text{Reshape}(s_{2})$$

(7) Y=GELU(F⊙A)

In Equation 6, **A** denotes the reshaped channel weights and is represented as $A\in {\mathbb{R}}^{1\times 1\times 3C_{\text{out}}}$. In Equation 7, the final output **Y** denotes the recalibrated feature map and is defined as $Y\in {\mathbb{R}}^{H\times W\times 3C_{\text{out}}}$.

#### 3.3.4 Loss function

The proposed model is optimized to minimize the cross-entropy loss in the learning process. Cross-entropy loss quantifies the difference between the predictive probability and actual labels and is defined as follows:

(8) $$Loss=-\frac{1}{N}\sum_{i=1}^{N}\sum_{c=1}^{C}y_{i,c}\text{log}({\hat{p}}_{i,c})$$

In Equation 8 N is the number of samples, C is the number of classes, $y_{i,c}$ is the one-hot real label, and ${\hat{p}}_{i,c}$ is the predictive probability.

### 3.4 Experimental configuration and training protocol

#### 3.4.1 Data splitting and cross-validation strategy

The submitted version of this work evaluated all models with shuffled stratified 3-fold cross-validation over images. Given the provenance structure reported in Section 3.2, that protocol places different photographs of the same plant on both sides of every split, and its scores are not estimates of generalisation to unseen plants. All results in this revision are therefore reported under grouped cross-validation, and the image-level protocol is retained only as an explicitly labelled leakage-positive reference so that the magnitude of the effect is visible rather than hidden.

Three grouping units are used, each with stratified 3-fold cross-validation and rotation of the test fold:

- Image-level (leakage-positive reference): shuffled folds over images, as in the submitted version.
- Plant-disjoint (primary protocol): stratified grouped folds [StratifiedGroupKFold (Pedregosa et al., 2011)] on the (greenhouse, plant) identifier, so that no plant contributes images to both the development and the test partition.
- Site-disjoint (strictest arm): folds grouped by greenhouse, so that neither the plant nor the acquisition site is shared. Because label and site are confounded in this corpus, this arm removes the site shortcut and the class-balanced signal together, and it is reported as a lower bound rather than as the headline result.

Validation data for model selection and early stopping are carved out of the development partition under the same grouping constraint as the outer split, so that a plant used for validation never appears in training. **Table 2** reports the realised leakage of each protocol, measured on the actual fold assignments rather than assumed from the design.

**Table 2:** Evaluation protocols and their realised leakage, measured on the actual fold assignments (mean over folds; 1.00 means every test-side group also appears in training).

Protocol	Grouping unit	Plant leakage	Site leakage	Date leakage	Test-fold size
Image-level (reference)	image	0.97	0.99–1.00	1.00	666–667
Plant-disjoint (primary)	(greenhouse, plant)	0.00	0.92–0.96	1.00	664–668
Site-disjoint (strictest)	greenhouse	0.00	0.00	1.00	658–674
Date-aware fold-design audits (restricted to the window in which both classes were acquired)					
Date-disjoint	acquisition date	0.91–0.96	0.97–0.98	0.00	296–679
Rolling-origin	acquisition date, forward	0.77–0.85	0.85–0.89	0.00	296–679
Plant × date	both	0.00	0.88–0.94	0.00	99–209

The plant-disjoint protocol is used for all results in Sections 4.3–4.11; the image-level protocol is reported only as a leakage-positive reference. Date-aware protocols are reported as fold-design audits.

Two further points are stated explicitly because they bound what the primary protocol can claim. First, plant grouping does not remove temporal overlap. Each plant was photographed hourly from July to December, so a plant-disjoint fold still contains the same acquisition dates on both sides: the measured date-leakage rate under the plant-disjoint protocol is 0.996. **Table 2** therefore also reports three date-aware protocols — date-disjoint, rolling-origin (every test image acquired strictly later than every training image) and combined plant × date — as fold-design audits. These protocols additionally have to be restricted to the calendar window in which both classes were acquired, since outside it the acquisition date is itself a perfect predictor of the label; that restriction discards 6 of 2,000 images for Tipburn, 605 for Fusarium wilt and 1,020 for Phytophthora blight, and leaves test partitions of 99–209 images in the strictest arm. Training under the date-aware protocols is reported as ongoing work rather than as a result, and no claim in this paper rests on it. Second, plant grouping does not remove the site confound, which is the reason for the site-disjoint arm.

#### 3.4.2 Classification models

The hyperparameter configurations of the classification models considered in this work are listed in **Table 3**. We evaluated MambaVision (Hatamizadeh and Kautz, 2025), NextViT (Li et al., 2022), ResNet (He et al., 2016), DenseNet (Huang et al., 2017), and EfficientNetV2 (Tan and Le, 2021). In addition, for MambaVision and EfficientNetV2, we constructed extended variants by incorporating the FDEA framework into the original backbones, denoted as MV-FDEA (Our Proposal 1) and EN-FDEA (Our Proposal 2), respectively, and compared their performance.

**Table 3:** Hyperparameter settings for the experimental models.

MambaVision-Tiny (Hatamizadeh and Kautz, 2025)		EfficientNetV2-S (Tan and Le, 2021)	
Dimension	80	Backbone dropout	0.2
Input dimension	32	Stochastic depth	0.2
Depths	(1,3,8,4)	Hidden dimension	512
Number of heads	(2,4,8,16)	Classifier dropout	0.1
Window size	(8,8,14,7)		
MLP ratio	4		
Drop path rate	0.2		
Hidden dimension	512		
Classifier dropout	0.1		

NextViT-Small (Li et al., 2022)		ResNet-50 (He et al., 2016)	
Stem channels	(64,32,64)	Feature dimension	2048
Depths	(3,4,10,3)	FC layers	(512,512,256)
Path dropout	0.1	FC stack dropout	0.5
Hidden dimension	512		
Classifier dropout	0.1		

DenseNet-121 (Huang et al., 2017)			
Feature dimension	1024		
FC layers	(512,512,256)		
FC stack dropout	0.5		

MV-FDEA (Our Prop. 1)		EN-FDEA (Our Prop. 2)	
(Backbone: MambaVision-Tiny)		(Backbone: EfficientNetV2-S)	
Dimension	80	Backbone dropout	0.2
Input dimension	32	Stochastic depth	0.2
Depths	(1,3,8,4)	Hidden dimension	512
Number of heads	(2,4,8,16)	Classifier dropout	0.1
Window size	(8,8,14,7)		
MLP ratio	4		
Drop path rate	0.2		
Hidden dimension	512		
FC stack dropout	0.5		

#### 3.4.3 Training strategy

All models were optimized with the Adam optimizer and Cross-Entropy loss using a batch size of 32 and a maximum of 50 epochs, with early stopping at a patience of 10 epochs on validation accuracy and restoration of the best-validation weights. Randomly initialised models used a learning rate of $1\times 10^{-3}$; ImageNet-initialised models were fine-tuned end to end at $1\times 10^{-4}$. Every configuration reported in Sections 4.3–4.11 was repeated over independent random seeds — five seeds (1007, 42, 2024, 7, 1234) × three folds = 15 runs for the principal comparison, and three seeds (1007, 42, 2024) × three folds = 9 runs for the initialisation and ablation studies — rather than the single seed used in the submitted version. We report the mean, the standard deviation over runs, the 95% interval over the run population, and, separately, the between-seed standard deviation of the fold-averaged score, which is the quantity that speaks to run-to-run stability.

#### 3.4.4 Hardware and implementation details

All experiments were implemented in PyTorch and executed on an NVIDIA RTX A6000 GPU. The submitted version trained every model from scratch and justified this as a way to assess the inherent feature-learning capability of each architecture. That justification is an assertion rather than evidence, and we withdraw it: initialisation is now treated as an experimental factor, with every architecture trained twice under identical splits and schedules (Section 4.5). Seeds are reported above; split definitions, per-image predictions and analysis scripts are released with the paper.

#### 3.4.5 Statistical analysis plan

Model-versus-model claims are supported by paired tests computed on identical test items rather than by overlapping confidence intervals. For each dataset and condition we compute McNemar’s test (McNemar, 1947) on paired predictions, as recommended for comparing classifiers on identical test items (Dietterich, 1998), and DeLong’s test for correlated ROC curves (DeLong et al., 1988) within each fold; over the paired seed × fold runs we compute a Wilcoxon signed-rank test with Cliff’s δ (Cliff, 1993) as effect size, following the recommendations of Demšar (2006) for comparisons across multiple datasets. Multiplicity is controlled with the Holm–Bonferroni procedure (Holm, 1979) across the full family of model comparisons, and effect sizes are reported alongside *p*-values throughout. The analysis plan was fixed before the comparisons were run, and the per-item predictions required to reproduce every test are released.

#### 3.4.6 Classification performance metrics

Balanced accuracy (BA) was adopted as the primary evaluation metric because it provides a balanced assessment under class-imbalanced settings by equally accounting for sensitivity and specificity. In addition, performance on the independent test set was measured using Loss, Precision, Recall, F1 score, and AUROC. To quantify statistical reliability, 95% confidence intervals for all evaluation metrics were estimated from 1,000 bootstrap resampling iterations. AUROC was further included to assess threshold-independent discriminative ability based on the trade-off between the true positive rate and false positive rate.

#### 3.4.7 Faithfulness metrics for visual explanations

To quantitatively assess the faithfulness of visual explanations produced by XAI methods, we employed two categories of perturbation-based metrics. First, average drop and increase in confidence proposed proposed by Chattopadhay et al. (2018) were used to examine confidence variation under single-step masking. However, because these single-step masking measures can be affected by factors such as mask generation strategy, threshold choice, and mask size, we additionally reported positive and negative perturbation AUC introduced by Chefer et al. (2021). Perturbation AUC evaluates the confidence-change trajectory by progressively inserting or removing input regions according to explanation importance, thereby offering a complementary perspective that is less sensitive to a single masking setup and better supports robust faithfulness evaluation.

Taken together, these complementary metrics enable a joint evaluation of classification performance, robustness under class imbalance, threshold-independent reliability, and faithfulness of visual explanations. Formal definitions of all evaluation metrics, including both classification and faithfulness metrics, are provided in Section S1 of the **Supplementary Material**.

## 4 Experiment results

### 4.1 Statistical and data visualization analysis

All exploratory analyses discussed in this section, including dimensionality reduction-based visualizations and feature distribution analyses, are provided in Section S2 of the **Supplementary Material**.

### 4.2 Overall classification performance under the image-level reference protocol

The results in this subsection reproduce the submitted version and are obtained under the image-level protocol, which Section 3.2 shows to place different photographs of the same plant on both sides of every fold. They are retained so that the effect of the protocol correction is visible, and they are labelled throughout as the leakage-positive reference. The results on which this paper’s conclusions rest begin in Section 4.3.

#### 4.2.1 Quantitative performance analysis of classification models

**Table 4** summarizes the averaged results of 3-fold cross-validation for the evaluated deep learning models on three strawberry disease datasets, namely Tipburn, Phytophthora blight, and Fusarium wilt, under the original and overexposure conditions, comparing the proposed models (MV-FDEA and EN-FDEA) with the baseline models. As a result of the experiment, EN-FDEA showed overall superior and stable classification performance compared with the baseline models, and this tendency was maintained for most disease types under the overexposure condition.

**Table 4:** Performance analysis of classification models under the image-level (leakage-positive) reference protocol, single seed.

Condition	Model	Balanced accuracy↑ (95% CI)	Loss↓ (95% CI)	Recall↑ (95% CI)	Precision↑ (95% CI)	F1 score↑ (95% CI)	AUROC↑ (95% CI)
Tipburn							
Original	MambaVision (Hatamizadeh and Kautz, 2025)	0.732 (0.710-0.753)	0.501 (0.477-0.526)	0.732 (0.711-0.752)	0.836 (0.816-0.855)	0.682 (0.653-0.713)	0.891 (0.868-0.912)
	NextViT (Li et al., 2022)	0.688 (0.667-0.710)	0.605 (0.576-0.634)	0.688 (0.667-0.711)	0.814 (0.794-0.834)	0.638 (0.604-0.672)	0.771 (0.740-0.803)
	EfficientNetV2 (Tan and Le, 2021)	0.695 (0.678-0.711)	0.546 (0.535-0.558)	0.695 (0.679-0.711)	0.649 (0.627-0.670)	0.626 (0.599-0.651)	0.732 (0.701-0.763)
	ResNet (He et al., 2016)	0.745 (0.723-0.766)	0.556 (0.538-0.575)	0.744 (0.724-0.765)	**0.842 (0.822-0.862)**	0.702 (0.674-0.732)	0.912 (0.893-0.930)
	DenseNet (Huang et al., 2017)	0.751 (0.726-0.775)	0.557 (0.539-0.575)	0.752 (0.727-0.777)	0.839 (0.819-0.859)	0.727 (0.695-0.759)	**0.947 (0.929-0.964)**
	MV-FDEA (our proposal 1)	0.687 (0.661-0.713)	0.779 (0.724-0.834)	0.687 (0.661-0.714)	0.806 (0.784-0.827)	0.652 (0.614-0.691)	0.907 (0.883-0.930)
	EN-FDEA (our proposal 2)	**0.793 (0.781-0.804)**	**0.367 (0.356-0.379)**	**0.792 (0.780-0.804)**	0.713 (0.696-0.731)	**0.738 (0.720-0.755)**	0.907 (0.888-0.925)
Overexposure	MambaVision (Hatamizadeh and Kautz, 2025)	0.746 (0.721-0.771)	0.485 (0.475-0.495)	0.746 (0.720-0.771)	0.828 (0.806-0.850)	0.723 (0.689-0.758)	0.856 (0.826-0.884)
	NextViT (Li et al., 2022)	0.768 (0.742-0.793)	0.473 (0.454-0.492)	0.767 (0.742-0.793)	0.841 (0.821-0.862)	0.750 (0.716-0.782)	0.910 (0.886-0.931)
	EfficientNetV2 (Tan and Le, 2021)	0.882 (0.859-0.905)	0.324 (0.300-0.347)	0.883 (0.860-0.905)	0.904 (0.885-0.924)	0.881 (0.857-0.906)	0.946 (0.930-0.960)
	ResNet (He et al., 2016)	0.830 (0.805-0.856)	0.480 (0.467-0.492)	0.830 (0.806-0.855)	0.873 (0.852-0.892)	0.824 (0.794-0.852)	0.963 (0.948-0.976)
	DenseNet (Huang et al., 2017)	0.802 (0.777-0.826)	0.512 (0.497-0.526)	0.802 (0.777-0.826)	0.858 (0.837-0.878)	0.788 (0.758-0.817)	0.921 (0.902-0.938)
	MV-FDEA (our proposal 1)	0.747 (0.721-0.774)	0.563 (0.531-0.596)	0.747 (0.722-0.774)	0.832 (0.811-0.852)	0.728 (0.692-0.763)	0.917 (0.893-0.937)
	EN-FDEA (our proposal 2)	**0.887 (0.865-0.908)**	**0.326 (0.311-0.342)**	**0.886 (0.865-0.908)**	**0.906 (0.887-0.924)**	0.882 (0.858-0.905)	**0.959 (0.944-0.972)**
Phytophthora blight							
Original	MambaVision (Hatamizadeh and Kautz, 2025)	0.878 (0.854-0.899)	0.350 (0.322-0.378)	0.878 (0.856-0.900)	0.906 (0.887-0.924)	0.875 (0.849-0.899)	0.990 (0.983-0.996)
	NextViT (Li et al., 2022)	0.751 (0.727-0.777)	1.170 (1.082-1.259)	0.751 (0.724-0.776)	0.837 (0.817-0.857)	0.728 (0.694-0.762)	0.895 (0.869-0.917)
	EfficientNetV2 (Tan and Le, 2021)	0.941 (0.925-0.956)	0.162 (0.150-0.173)	0.941 (0.925-0.956)	0.948 (0.934-0.962)	0.939 (0.922-0.955)	0.991 (0.984-0.996)
	ResNet (He et al., 2016)	0.900 (0.879-0.920)	0.407 (0.399-0.414)	0.900 (0.879-0.920)	0.916 (0.898-0.934)	0.898 (0.874-0.921)	0.994 (0.988-0.998)
	DenseNet (Huang et al., 2017)	0.862 (0.847-0.876)	0.447 (0.437-0.458)	0.862 (0.848-0.877)	0.919 (0.906-0.931)	0.843 (0.823-0.862)	0.880 (0.865-0.895)
	MV-FDEA (our proposal 1)	0.944 (0.928-0.960)	0.150 (0.138-0.163)	0.944 (0.928-0.960)	0.950 (0.935-0.964)	0.943 (0.926-0.959)	**0.997 (0.996-0.999)**
	EN-FDEA (our proposal 2)	**0.953 (0.938-0.967)**	**0.125 (0.116-0.133)**	**0.953 (0.939-0.967)**	**0.959 (0.945-0.972)**	**0.954 (0.938-0.968)**	0.996 (0.993-0.999)
Overexposure	MambaVision (Hatamizadeh and Kautz, 2025)	0.916 (0.895-0.936)	0.209 (0.195-0.223)	0.916 (0.895-0.935)	0.928 (0.911-0.946)	0.915 (0.894-0.935)	0.980 (0.970-0.988)
	NextViT (Li et al., 2022)	0.812 (0.788-0.835)	0.456 (0.421-0.490)	0.812 (0.789-0.834)	0.873 (0.853-0.892)	0.795 (0.765-0.822)	0.965 (0.955-0.975)
	EfficientNetV2 (Tan and Le, 2021)	**0.990 (0.982-0.996)**	**0.048 (0.044-0.053)**	**0.990 (0.982-0.996)**	**0.989 (0.982-0.996)**	**0.989 (0.981-0.996)**	0.965 (0.955-0.975)
	ResNet (He et al., 2016)	0.942 (0.925-0.958)	0.368 (0.363-0.373)	0.942 (0.925-0.958)	0.949 (0.934-0.964)	0.942 (0.924-0.959)	0.997 (0.993-0.998)
	DenseNet (Huang et al., 2017)	0.956 (0.941-0.971)	0.355 (0.352-0.359)	0.956 (0.940-0.971)	0.960 (0.945-0.973)	0.956 (0.940-0.971)	**0.997 (0.994-0.999)**
	MV-FDEA (our proposal 1)	0.766 (0.742-0.790)	1.313 (1.214-1.418)	0.766 (0.741-0.792)	0.847 (0.827-0.867)	0.745 (0.711-0.776)	0.993 (0.988-0.997)
	EN-FDEA (our proposal 2)	0.944 (0.929-0.958)	0.209 (0.191-0.227)	0.945 (0.929-0.958)	0.954 (0.941-0.967)	0.944 (0.928-0.959)	0.985 (0.976-0.992)
Fusarium wilt							
Original	MambaVision (Hatamizadeh and Kautz, 2025)	0.880 (0.860-0.901)	0.460 (0.426-0.494)	0.881 (0.859-0.900)	0.909 (0.891-0.926)	0.876 (0.852-0.899)	0.951 (0.933-0.966)
	Fusarium wilt (continued)							
	NextViT (Li et al., 2022)	0.847 (0.825-0.868)	0.549 (0.507-0.592)	0.847 (0.825-0.868)	0.887 (0.868-0.905)	0.839 (0.813-0.864)	0.980 (0.970-0.987)
	EfficientNetV2 (Tan and Le, 2021)	0.827 (0.806-0.848)	0.803 (0.744-0.861)	0.827 (0.806-0.848)	0.881 (0.863-0.899)	0.811 (0.785-0.836)	0.889 (0.866-0.909)
	ResNet (He et al., 2016)	0.956 (0.939-0.970)	0.356 (0.352-0.358)	0.956 (0.940-0.970)	0.957 (0.942-0.972)	0.956 (0.940-0.971)	0.992 (0.986-0.997)
	DenseNet (Huang et al., 2017)	0.884 (0.862-0.906)	0.422 (0.413-0.430)	0.884 (0.862-0.907)	0.903 (0.884-0.922)	0.882 (0.856-0.907)	0.966 (0.956-0.976)
	MV-FDEA (our proposal 1)	0.814 (0.789-0.839)	0.504 (0.464-0.545)	0.813 (0.788-0.838)	0.867 (0.846-0.887)	0.806 (0.776-0.835)	0.993 (0.988-0.997)
EN-FDEA (our proposal 2)	**0.976 (0.965-0.987)**	**0.072 (0.066-0.078)**	**0.976 (0.965-0.987)**	**0.977 (0.967-0.987)**	**0.977 (0.964-0.986)**	**0.998 (0.995-0.999)**
Overexposure	MambaVision (Hatamizadeh and Kautz, 2025)	0.678 (0.653-0.702)	1.415 (1.306-1.525)	0.677 (0.652-0.702)	0.806 (0.785-0.826)	0.633 (0.597-0.670)	0.936 (0.919-0.951)
	NextViT (Li et al., 2022)	0.686 (0.666-0.705)	1.766 (1.632-1.905)	0.686 (0.667-0.705)	0.818 (0.796-0.837)	0.620 (0.589-0.652)	0.825 (0.803-0.846)
	EfficientNetV2 (Tan and Le, 2021)	0.808 (0.790-0.826)	0.770 (0.712-0.828)	0.808 (0.789-0.825)	0.884 (0.867-0.899)	0.780 (0.754-0.805)	0.795 (0.775-0.814)
	ResNet (He et al., 2016)	0.818 (0.804-0.831)	0.493 (0.479-0.505)	0.818 (0.805-0.830)	0.896 (0.881-0.911)	0.773 (0.753-0.792)	0.975 (0.965-0.982)
	DenseNet (Huang et al., 2017)	0.764 (0.748-0.780)	0.538 (0.521-0.555)	0.765 (0.749-0.780)	0.864 (0.846-0.882)	0.713 (0.691-0.734)	0.922 (0.909-0.934)
	MV-FDEA(our proposal 1)	0.830 (0.805-0.856)	0.356 (0.330-0.383)	0.830 (0.806-0.855)	0.873 (0.854-0.894)	0.825 (0.794-0.854)	0.993 (0.989-0.997)
	EN-FDEA(our proposal 2)	**0.995 (0.991-0.998)**	**0.027 (0.025-0.030)**	**0.995 (0.991-0.998)**	**0.995 (0.991-0.998)**	**0.995 (0.991-0.998)**	**0.998 (0.995-1.000)**

These values are not estimates of generalisation to unseen plants; see **Table 5** for the corrected protocol.Bold values indicate the best value of each metric within each dataset and condition.

For Tipburn, the balanced accuracy of the baseline models ranged from 0.69 to 0.75, suggesting that this disease type is relatively difficult to classify because of its subtle and low-contrast lesion characteristics. Under the original condition, EN-FDEA achieved a balanced accuracy of 0.793, showing the best performance among the evaluated models and exceeding the strongest baseline by 0.042. In addition, compared with its backbone baseline, EfficientNetV2, EN-FDEA improved balanced accuracy by 0.098 and reduced loss by 0.179, corresponding to a 14.10% improvement in balanced accuracy. Under the overexposure condition, EN-FDEA also achieved the highest balanced accuracy of 0.887 among the baseline and proposed models, showing a 0.57% improvement over the backbone baseline.

For Phytophthora blight, EN-FDEA achieved the highest performance under the original condition, with a balanced accuracy of 0.953. Compared with the backbone baseline, EfficientNetV2, this corresponds to an improvement of 0.012 in balanced accuracy, or 1.28%. In contrast, under the overexposure condition, EN-FDEA maintained relatively high performance with a balanced accuracy of 0.944 and an F1-score of 0.944, although its performance was slightly lower than that of DenseNet and EfficientNetV2. In particular, EfficientNetV2 achieved the highest performance with a balanced accuracy of 0.990 and an F1-score of 0.989, while EN-FDEA showed a 4.65% decrease relative to the backbone baseline. These findings suggest that, for Phytophthora blight, the benefit of the proposed feature enhancement strategy under the overexposure condition may be more limited than for the other disease types.

For Fusarium wilt, EN-FDEA showed the best classification performance under both the original and overexposure conditions. Under the original condition, EN-FDEA achieved a balanced accuracy of 0.976, corresponding to an 18.02% improvement over the backbone baseline, EfficientNetV2. Under the overexposure condition, EN-FDEA further achieved a balanced accuracy of 0.995, showing a 23.14% improvement over the backbone baseline. Notably, whereas the baseline models generally showed performance degradation when transitioning from the original to the overexposure condition, the proposed model instead showed an increasing trend in balanced accuracy. These results suggest that, for Fusarium wilt, the proposed FDEA module can effectively preserve and enhance lesion-related features even in the presence of visual distortion caused by overexposure.

Under this protocol EN-FDEA improved balanced accuracy by an average of 11.13% over the backbone baseline in the original condition and 6.35% under overexposure. Both figures are withdrawn: Section 4.4 shows that they do not survive the removal of plant leakage and the addition of seed replication, and Section 4.10 reports that seventeen of eighteen paired comparisons are non-significant after multiplicity correction. They are reproduced here only to quantify what the protocol correction changes.

#### 4.2.2 Qualitative analysis of classification performance

**Figure 3** presents the ROC and PR curves for Tipburn, Phytophthora blight, and Fusarium wilt under the original and overexposure conditions, allowing a qualitative comparison of the discriminative capability and robustness of the evaluated models.

**Figure 3:**
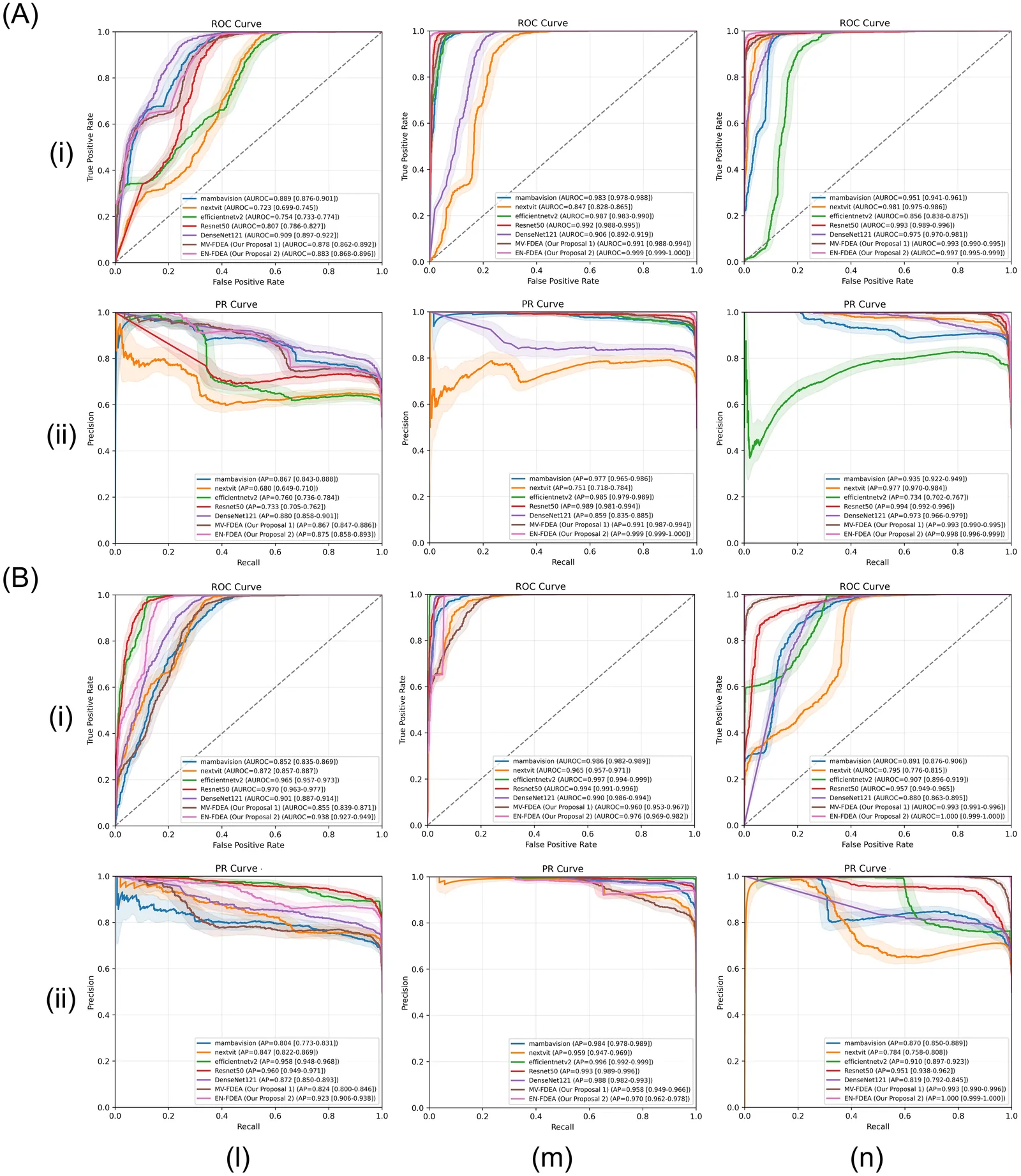
Qualitative comparison of model performance using ROC and PR curves. **(A)** Original condition, **(B)** Overexposure condition, **(l)** Tipburn, **(m)** Phytophthora blight, **(n)** Fusarium wilt, **(i)** ROC curve, and **(ii)** PR curve.

In the ROC analysis shown in **Figure 3(i)**, the proposed models, EN-FDEA and MV-FDEA, generally exhibited more favorable or competitive curve patterns than the baseline models, with this tendency being more pronounced for Phytophthora blight and Fusarium wilt. These results suggest that the proposed framework can effectively distinguish normal leaf tissues from low-contrast early lesion patterns. In contrast, for Tipburn, the ROC curves of all models were relatively less steep than those for the other disease types, indicating that this disease type is more difficult to classify.

In the PR analysis shown in **Figure 3(ii)**, the baseline models tended to show a relatively rapid decline in precision at higher recall levels for Tipburn, where early micro-lesions are visually mixed with normal leaf textures. By contrast, EN-FDEA maintained more stable precision, indicating its effectiveness in capturing fine-grained lesion features. For Fusarium wilt, the proposed models also maintained overall high precision-recall curves, indicating strong classification reliability.

Comparing **Figure 3(A)** and **Figure 3(B)**, the proposed models generally showed more favorable ROC and PR curve patterns than the baseline models in the original condition. Under the overexposure condition, however, the differences between the proposed and baseline models became less pronounced for Tipburn and Phytophthora blight, resulting in more similar overall curve patterns. Nevertheless, for Fusarium wilt, EN-FDEA still maintained the most favorable curve patterns even under the overexposure condition. These findings indicate that the effectiveness of the proposed FDEA-based feature enhancement structure may vary depending on the disease type and environmental condition, while its advantage remains particularly evident for Fusarium wilt under visually degraded conditions.

#### 4.2.3 Calibration analysis for reliable decision-making

To examine the reliability of the probability outputs of EN-FDEA, calibration plots were analyzed under the original and overexposure conditions, as shown in **Figure 4**.

**Figure 4:**
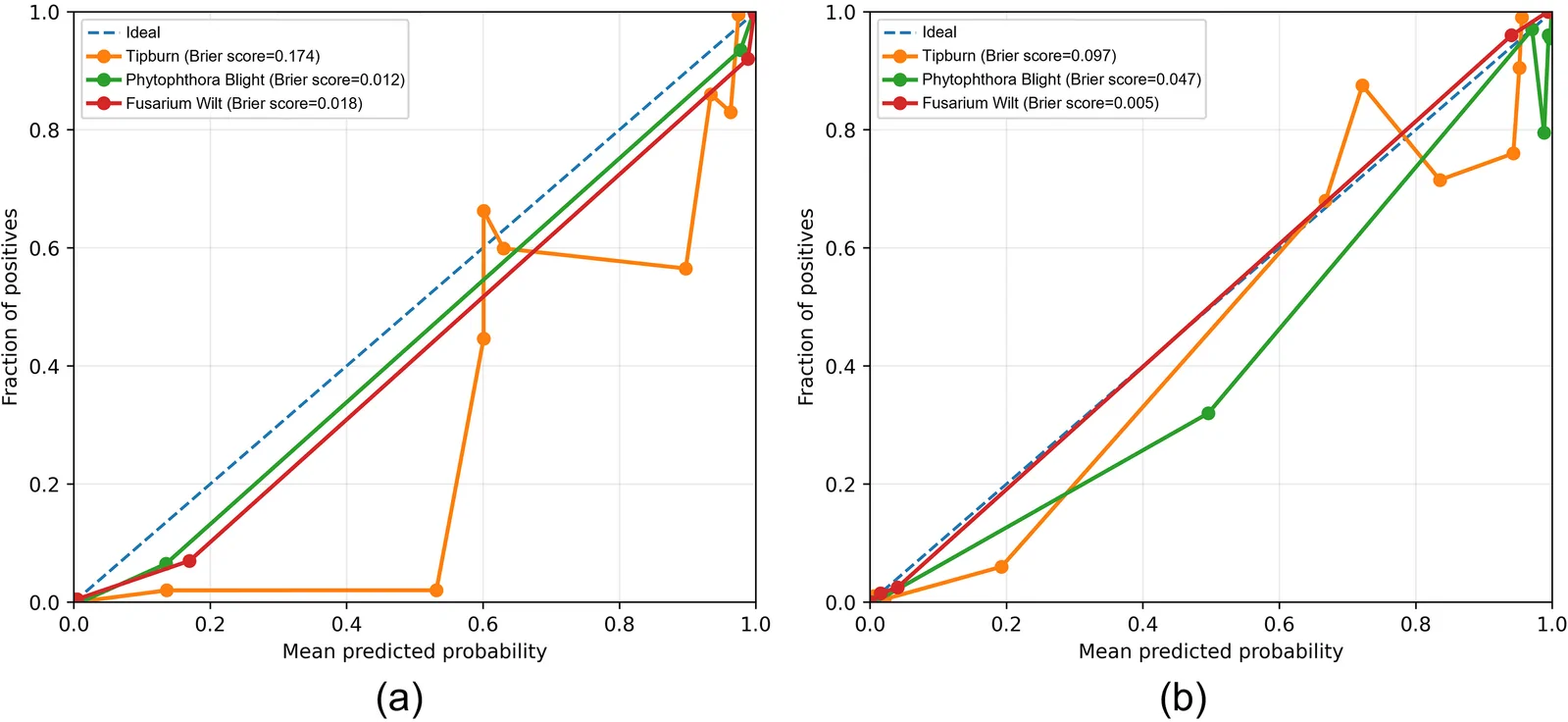
Calibration plots for the EN-FDEA, **(a)** Original, and **(b)** Overexposure.

**Figure 4a** presents the calibration results under the original condition. EN-FDEA showed favorable calibration performance for Phytophthora blight and Fusarium wilt, with low Brier scores of 0.012 and 0.018, respectively. In contrast, Tipburn recorded a relatively higher Brier score of 0.174, indicating lower calibration performance than the other disease types. This may be attributed to the visual ambiguity and low-contrast characteristics of early micro-lesions mixed with the natural venation structure of the leaves, and some degree of overconfidence was observed in certain probability ranges.

**Figure 4b** shows the calibration results under the overexposure condition. The Brier scores for Phytophthora blight and Fusarium wilt remained low at 0.047 and 0.005, respectively, whereas Tipburn recorded 0.097. However, the calibration curves showed different patterns across disease types. Tipburn exhibited a relatively higher Brier score, but its curve was irregularly distributed around the ideal diagonal without a clearly consistent tendency toward overconfidence or underconfidence. In contrast, although Phytophthora blight maintained a low overall Brier score, overconfidence was observed in the mid-probability range, where the curve fell below the ideal diagonal. Fusarium wilt showed the best calibration characteristics, with the curve remaining closest to the ideal diagonal across the full probability range. These results suggest that the predicted probabilities of EN-FDEA remained generally stable under the overexposure condition, although the calibration characteristics varied depending on the disease type and probability range.

### 4.3 Effect of the evaluation protocol on measured performance

**Table 5** isolates the contribution of the evaluation protocol by training the same architectures under identical schedules and varying only the grouping unit. Enforcing plant disjointness costs between 0.002 and 0.082 balanced accuracy, with the largest losses on Tipburn, the dataset whose lesions are least distinct. Enforcing site disjointness costs substantially more: EN-FDEA falls by 0.144 on Tipburn and by 0.173 on Phytophthora blight, and the Phytophthora blight result in particular shows that a large part of the near-ceiling image-level score was attributable to greenhouse identity rather than to pathology. Fusarium wilt is the least affected dataset in both directions, consistent with its lesions producing morphological change that survives a change of site.

**Table 5:** Balanced accuracy under the three grouping protocols (3 folds, seed 1007).

Dataset	Condition	Model	Image-level	Plant-disjoint	Site-disjoint	$\Delta_{\text{plant}}$	$\Delta_{\text{site}}$
Tipburn	Original	EN-FDEA	0.959	0.900	0.815	–0.059	–0.144
		EfficientNetV2	0.947	0.908	0.878	–0.039	–0.069
	Overexposure	EN-FDEA	0.900	0.862	0.809	–0.038	–0.091
		EfficientNetV2	0.928	0.846	0.812	–0.082	–0.116
Phytophthora blight	Original	EN-FDEA	0.995	0.989	0.821	–0.006	–0.173
		EfficientNetV2	0.987	0.980	0.966	–0.007	–0.021
	Overexposure	EN-FDEA	0.993	0.991	0.979	–0.002	–0.015
		EfficientNetV2	0.991	0.980	0.965	–0.012	–0.026
Fusarium wilt	Original	EN-FDEA	0.991	0.986	0.972	–0.005	–0.020
		EfficientNetV2	0.990	0.988	0.988	–0.002	–0.002
	Overexposure	EN-FDEA	0.990	0.983	0.951	–0.007	–0.039
		EfficientNetV2	0.989	0.978	0.941	–0.011	–0.048

The image-level column reproduces the protocol used in the submitted version and is reported only as a leakage-positive reference. $\Delta_{\text{plant}}$ and $\Delta_{\text{site}}$ are the changes relative to the image-level reference.

Two consequences follow for the rest of this paper. All results reported below use the plant-disjoint protocol, which is leakage-free with respect to plants while retaining a test partition large enough to support comparison. The site-disjoint arm is reported as a bound rather than as the primary result, because in a corpus where site and label are perfectly confounded it removes the label-correlated site signal and the site shortcut simultaneously, and cannot separate them.

### 4.4 Seed-level stability and comparison with baselines

**Table 6** reports the principal comparison under the primary protocol. The picture it gives differs materially from the single-seed, image-level result of the submitted version, and we state the difference plainly. Under leakage-free evaluation, EN-FDEA is not better than its EfficientNetV2 backbone on the original condition of any dataset: the mean differences are –0.013 (Tipburn), –0.002 (Phytophthora blight) and –0.002 (Fusarium wilt), all smaller than the corresponding run-to-run spread. On the overexposure condition EN-FDEA is ahead of the backbone on Phytophthora blight (+0.012) and Fusarium wilt (+0.005) and behind on Tipburn (–0.005). The strongest overall results on Phytophthora blight and Fusarium wilt under the original condition belong to DenseNet121, not to the proposed model.

**Table 6:** Balanced accuracy under the plant-disjoint protocol over five seeds × three folds (15 runs per cell).

Dataset	Model	Original		Overexposure	
		BA (mean ± SD)	Between-seed SD	BA (mean ± SD)	Between-seed SD
Tipburn	EN-FDEA	0.895 ± 0.031	0.010	0.856 ± 0.046	0.026
	EfficientNetV2	**0.908** ± **0.028**	0.013	0.861 ± 0.039	0.017
	ResNet50	0.896 ± 0.039	0.026	**0.878** ± **0.042**	0.019
	DenseNet121	0.895 ± 0.047	0.040	0.854 ± 0.102	0.063
Phytophthora blight	EN-FDEA	0.984 ± 0.014	0.008	**0.988** ± **0.009**	0.004
	EfficientNetV2	0.986 ± 0.007	0.004	0.976 ± 0.016	0.005
	ResNet50	0.988 ± 0.007	0.003	0.976 ± 0.013	0.007
	DenseNet121	**0.990** ± **0.006**	0.003	0.985 ± 0.009	0.005
Fusarium wilt	EN-FDEA	0.987 ± 0.009	0.006	**0.985** ± **0.010**	0.006
	EfficientNetV2	0.989 ± 0.006	0.002	0.980 ± 0.012	0.002
	ResNet50	0.985 ± 0.009	0.004	0.982 ± 0.007	0.006
	DenseNet121	**0.991** ± **0.005**	0.001	0.978 ± 0.013	0.006

SD is over the 15 runs; between-seed SD is the standard deviation of the fold-averaged score across seeds and is the quantity relevant to run-to-run stability.Bold values indicate the highest mean balanced accuracy within each dataset and condition.

The between-seed standard deviations explain why the submitted single-seed comparison was not reliable. On Tipburn the fold-averaged score of DenseNet121 varies by 0.040 across seeds under the original condition and by 0.063 under overexposure, which is several times the margins the submitted version reported as evidence of superiority. A single run of two models on this corpus can order them either way. The averages in **Table 6** are stable in a way single runs are not, and the paired tests of Section 4.10 are the basis on which we now make or withhold any claim of superiority.

### 4.5 Initialisation: random versus ImageNet

**Table 7** answers two questions at once: whether from-scratch training was a defensible default, and how the proposed model compares against strong pretrained baselines rather than against a training setup of our own choosing. ImageNet initialisation improves every architecture on every dataset, by 0.037–0.060 for the convolutional models and by 0.34–0.50 for the two transformer baselines, which do not train usefully from scratch on 2,000 images and collapse to chance on most datasets. The from-scratch protocol of the submitted version therefore did not measure inherent feature-learning capability; it measured which architectures tolerate small-sample training, and it systematically disadvantaged the transformer baselines against which the proposed model was compared.

**Table 7:** Balanced accuracy by initialisation under the plant-disjoint protocol, original condition, three seeds × three folds.

Model	Tipburn		Phytophthora blight		Fusarium wilt	
	Scratch	ImageNet	Scratch	ImageNet	Scratch	ImageNet
EN-FDEA	0.891	0.948	0.986	0.994	0.988	0.998
EfficientNetV2	0.915	0.952	0.984	1.000	0.988	0.999
ResNet50	0.893	0.953	0.988	1.000	0.983	0.999
DenseNet121	0.890	0.950	0.989	1.000	0.991	1.000
ConvNeXt-T	0.500	0.957	0.597	1.000	0.656	1.000
Swin-T	0.500	0.962	0.500	0.999	0.500	0.999

Both arms use identical splits, schedules and early stopping; scratch models are trained at $1\times 10^{-3}$ and ImageNet models fine-tuned end to end at $1\times 10^{-4}$.

Under the pretrained setting, which is the configuration a practitioner would deploy, the differences between architectures largely disappear: on Phytophthora blight and Fusarium wilt every model reaches 0.994–1.000, and on Tipburn the range is 0.948–0.962 with EN-FDEA at the bottom of that range. We do not read these near-ceiling values as evidence that the diagnostic problem is solved; Section 4.3 shows that a large part of them is site signature, and Section 4.9 bounds how much.

### 4.6 FDEA module analysis

#### 4.6.1 Effect analysis of the FDEA module configurations

To optimize the structural design of the FDEA module, we conducted an effectiveness analysis of three major components: the number of parallel branches in the Parallel Feature Amplification stage, the branch dilation-rate configuration, and the activation function used in the channel recalibration stage. **Table 8** presents the quantitative performance variations across these configurations under the original and overexposure conditions.

**Table 8:** Effect analysis of the FDEA module under the image-level (leakage-positive) reference protocol, single seed.

Factor	Configuration	Condition	Balanced accuracy↑ (95% CI)	Loss↓ (95% CI)	Recall↑ (95% CI)	Precision↑ (95% CI)	F1 score↑ (95% CI)	AUROC↑ (95% CI)
Parallel branch number	Tipburn							
	1	Original	0.647 (0.641-0.652)	0.541 (0.527-0.557)	0.647 (0.641-0.653)	0.480 (0.461-0.498)	0.535 (0.517-0.552)	0.843 (0.816-0.868)
	2		0.766 (0.751-0.780)	0.412 (0.401-0.423)	0.766 (0.751-0.780)	0.692 (0.672-0.712)	0.711 (0.690-0.730)	0.909 (0.888-0.928)
	3		0.901 (0.879-0.921)	0.267 (0.258-0.275)	0.901 (0.880-0.921)	0.915 (0.895-0.934)	0.900 (0.876-0.922)	0.970 (0.957-0.982)
	1	Overexposure	0.875 (0.853-0.896)	0.332 (0.314-0.351)	0.875 (0.852-0.897)	0.902 (0.882-0.921)	0.873 (0.847-0.896)	0.964 (0.949-0.977)
	2		0.893 (0.873-0.913)	0.247 (0.234-0.261)	0.893 (0.872-0.914)	0.914 (0.895-0.932)	0.892 (0.868-0.914)	0.977 (0.966-0.985)
	3		0.721 (0.706-0.736)	0.462 (0.452-0.472)	0.721 (0.706-0.736)	0.659 (0.637-0.680)	0.658 (0.635-0.681)	0.795 (0.767-0.821)
	Phytophthora blight							
	1	Original	0.814 (0.805-0.821)	0.312 (0.299-0.325)	0.814 (0.805-0.822)	0.730 (0.716-0.743)	0.755 (0.742-0.769)	0.925 (0.910-0.938)
	2		0.638 (0.632-0.645)	0.563 (0.552-0.574)	0.639 (0.632-0.645)	0.477 (0.458-0.496)	0.529 (0.510-0.546)	0.764 (0.740-0.787)
	3		0.979 (0.967-0.988)	0.078 (0.072-0.084)	0.979 (0.968-0.988)	0.978 (0.967-0.988)	0.978 (0.967-0.988)	0.997 (0.992-1.000)
	1	Overexposure	0.950 (0.938-0.961)	0.107 (0.100-0.114)	0.950 (0.938-0.961)	0.957 (0.946-0.967)	0.947 (0.934-0.960)	0.997 (0.996-0.999)
	2		0.952 (0.938-0.965)	0.128 (0.122-0.134)	0.952 (0.938-0.964)	0.960 (0.948-0.971)	0.953 (0.937-0.966)	0.996 (0.994-0.998)
	3		0.822 (0.816-0.829)	0.263 (0.254-0.272)	0.823 (0.816-0.828)	0.742 (0.729-0.754)	0.769 (0.757-0.780)	0.969 (0.960-0.977)
	Fusarium wilt							
	1	Original	0.989 (0.982-0.996)	0.039 (0.035-0.044)	0.989 (0.982-0.996)	0.990 (0.982-0.996)	0.989 (0.981-0.996)	0.998 (0.995-1.000)
	2		0.951 (0.936-0.965)	0.145 (0.135-0.155)	0.951 (0.936-0.965)	0.956 (0.943-0.969)	0.951 (0.935-0.965)	0.995 (0.991-0.998)
	3		0.967 (0.953-0.980)	0.129 (0.117-0.140)	0.967 (0.953-0.980)	0.970 (0.957-0.982)	0.968 (0.954-0.980)	0.996 (0.993-0.999)
	1	Overexposure	0.931 (0.912-0.949)	0.192 (0.178-0.207)	0.931 (0.912-0.949)	0.939 (0.921-0.955)	0.930 (0.910-0.948)	0.996 (0.994-0.998)
	2		0.965 (0.951-0.978)	0.107 (0.098-0.116)	0.965 (0.951-0.978)	0.967 (0.953-0.979)	0.965 (0.950-0.978)	0.998 (0.996-1.000)
	3		0.966 (0.951-0.979)	0.112 (0.104-0.120)	0.966 (0.952-0.978)	0.968 (0.956-0.980)	0.966 (0.952-0.979)	0.996 (0.992-0.999)
	Average							
	1	–	0.868 (0.855-0.879)	0.254 (0.242-0.266)	0.868 (0.855-0.880)	0.833 (0.818-0.847)	0.838 (0.822-0.854)	**0.954 (0.943-0.963)**
	2		0.861 (0.847-0.874)	0.267 (0.257-0.277)	0.861 (0.847-0.874)	0.828 (0.812-0.843)	0.833 (0.815-0.850)	0.940 (0.929-0.949)
	3		**0.893 (0.879-0.906)**	**0.219 (0.210-0.227)**	**0.893 (0.879-0.905)**	**0.872 (0.857-0.886)**	**0.873 (0.857-0.888)**	**0.954 (0.944-0.963)**
Branch dilation-rate configuration	Tipburn							
	1,1,1	Original	0.901 (0.879-0.921)	0.267 (0.258-0.275)	0.901 (0.880-0.921)	0.915 (0.895-0.934)	0.900 (0.876-0.922)	0.970 (0.957-0.982)
	1,2,4		0.875 (0.854-0.896)	0.370 (0.346-0.393)	0.875 (0.853-0.895)	0.905 (0.887-0.923)	0.871 (0.845-0.894)	0.955 (0.940-0.968)
	2,4,8		0.778 (0.753-0.804)	0.470 (0.455-0.485)	0.779 (0.753-0.803)	0.846 (0.823-0.867)	0.760 (0.727-0.791)	0.909 (0.887-0.929)
	1,1,1	Overexposure	0.721 (0.706-0.736)	0.462 (0.452-0.472)	0.721 (0.706-0.736)	0.659 (0.637-0.680)	0.658 (0.635-0.681)	0.795 (0.767-0.821)
	1,2,4		0.733 (0.717-0.748)	0.491 (0.478-0.505)	0.733 (0.716-0.749)	0.669 (0.649-0.689)	0.673 (0.648-0.696)	0.858 (0.830-0.883)
	2,4,8		0.777 (0.763-0.790)	0.387 (0.379-0.396)	0.776 (0.762-0.790)	0.698 (0.679-0.716)	0.718 (0.698-0.737)	0.893 (0.872-0.914)
	Phytophthora blight							
	1,1,1	Original	0.979 (0.967-0.988)	0.078 (0.072-0.084)	0.979 (0.968-0.988)	0.978 (0.967-0.988)	0.978 (0.967-0.988)	0.997 (0.992-1.000)
	1,2,4		0.963 (0.950-0.975)	0.111 (0.102-0.121)	0.963 (0.950-0.975)	0.965 (0.953-0.976)	0.962 (0.949-0.975)	0.994 (0.990-0.998)
	2,4,8		0.922 (0.904-0.937)	0.215 (0.197-0.231)	0.921 (0.903-0.939)	0.937 (0.922-0.952)	0.922 (0.902-0.940)	0.990 (0.984-0.994)
	1,1,1	Overexposure	0.822 (0.816-0.829)	0.263 (0.254-0.272)	0.823 (0.816-0.828)	0.742 (0.729-0.754)	0.769 (0.757-0.780)	0.969 (0.960-0.977)
	1,2,4		0.780 (0.768-0.790)	0.406 (0.384-0.427)	0.779 (0.769-0.789)	0.711 (0.696-0.727)	0.725 (0.707-0.741)	0.966 (0.958-0.974)
	2,4,8		0.941 (0.927-0.954)	0.181 (0.168-0.197)	0.940 (0.926-0.954)	0.954 (0.941-0.965)	0.940 (0.925-0.955)	0.988 (0.982-0.993)
	Fusarium wilt							
	1,1,1	Original	0.967 (0.953-0.980)	0.129 (0.117-0.140)	0.967 (0.953-0.980)	0.970 (0.957-0.982)	0.968 (0.954-0.980)	0.996 (0.993-0.999)
	1,2,4		0.949 (0.934-0.963)	0.164 (0.151-0.176)	0.949 (0.934-0.964)	0.958 (0.945-0.969)	0.949 (0.934-0.963)	0.997 (0.993-0.999)
	2,4,8		0.737 (0.724-0.750)	0.523 (0.498-0.546)	0.737 (0.725-0.749)	0.687 (0.669-0.703)	0.677 (0.656-0.697)	0.950 (0.936-0.962)
	1,1,1	Overexposure	0.966 (0.951-0.979)	0.112 (0.104-0.120)	0.966 (0.952-0.978)	0.968 (0.956-0.980)	0.966 (0.952-0.979)	0.996 (0.992-0.999)
	1,2,4		0.946 (0.932-0.959)	0.135 (0.126-0.144)	0.946 (0.932-0.959)	0.956 (0.944-0.969)	0.946 (0.931-0.960)	0.986 (0.978-0.992)
	2,4,8		0.911 (0.893-0.928)	0.435 (0.400-0.471)	0.911 (0.894-0.927)	0.935 (0.920-0.949)	0.910 (0.890-0.927)	0.988 (0.982-0.994)
	Average							
	1,1,1	–	**0.893 (0.879-0.906)**	**0.219 (0.210-0.227)**	**0.893 (0.879-0.905)**	**0.872 (0.857-0.886)**	**0.873 (0.857-0.888)**	0.954 (0.944-0.963)
	1,2,4		0.874 (0.859-0.888)	0.280 (0.265-0.294)	0.874 (0.859-0.888)	0.861 (0.846-0.876)	0.854 (0.836-0.872)	**0.959 (0.948-0.969)**
	2,4,8		0.844 (0.827-0.861)	0.369 (0.349-0.388)	0.844 (0.827-0.860)	0.843 (0.826-0.859)	0.821 (0.800-0.841)	0.953 (0.941-0.964)
Channel recalibration activation	Tipburn							
	sigmoid	Original	0.872 (0.849-0.894)	0.356 (0.346-0.365)	0.871 (0.848-0.893)	0.890 (0.869-0.911)	0.868 (0.842-0.892)	0.940 (0.920-0.956)
	logsigmoid		0.901 (0.879-0.921)	0.267 (0.258-0.275)	0.901 (0.880-0.921)	0.915 (0.895-0.934)	0.900 (0.876-0.922)	0.970 (0.957-0.982)
	sigmoid	Overexposure	0.733 (0.716-0.748)	0.466 (0.453-0.480)	0.733 (0.717-0.748)	0.671 (0.652-0.690)	0.673 (0.649-0.695)	0.896 (0.872-0.919)
	logsigmoid		0.721 (0.706-0.736)	0.462 (0.452-0.472)	0.721 (0.706-0.736)	0.659 (0.637-0.680)	0.658 (0.635-0.681)	0.795 (0.767-0.821)
	Phytophthora blight							
	sigmoid	Original	0.806 (0.796-0.815)	0.375 (0.354-0.397)	0.806 (0.796-0.815)	0.722 (0.707-0.737)	0.748 (0.731-0.762)	0.934 (0.923-0.946)
	logsigmoid		0.979 (0.967-0.988)	0.078 (0.072-0.084)	0.979 (0.968-0.988)	0.978 (0.967-0.988)	0.978 (0.967-0.988)	0.997 (0.992-1.000)
	sigmoid	Overexposure	0.953 (0.936-0.967)	0.161 (0.148-0.173)	0.953 (0.936-0.968)	0.957 (0.942-0.971)	0.953 (0.936-0.968)	0.992 (0.986-0.997)
	logsigmoid		0.822 (0.816-0.829)	0.263 (0.254-0.272)	0.823 (0.816-0.828)	0.742 (0.729-0.754)	0.769 (0.757-0.780)	0.969 (0.960-0.977)
	Fusarium wilt							
	sigmoid	Original	0.977 (0.966-0.987)	0.063 (0.058-0.068)	0.977 (0.965-0.987)	0.978 (0.968-0.989)	0.978 (0.966-0.988)	0.998 (0.996-1.000)
	logsigmoid		0.967 (0.953-0.980)	0.129 (0.117-0.140)	0.967 (0.953-0.980)	0.970 (0.957-0.982)	0.968 (0.954-0.980)	0.996 (0.993-0.999)
	sigmoid	Overexposure	0.974 (0.961-0.985)	0.078 (0.071-0.085)	0.974 (0.961-0.985)	0.975 (0.963-0.986)	0.974 (0.961-0.985)	0.999 (0.997-1.000)
	logsigmoid		0.966 (0.951-0.979)	0.112 (0.104-0.120)	0.966 (0.952-0.978)	0.968 (0.956-0.980)	0.966 (0.952-0.979)	0.996 (0.992-0.999)
	Average							
	sigmoid	–	0.886 (0.871-0.899)	0.250 (0.238-0.261)	0.886 (0.870-0.899)	0.865 (0.850-0.881)	0.866 (0.848-0.882)	**0.960 (0.949-0.970)**
	logsigmoid		**0.893 (0.879-0.906)**	**0.219 (0.210-0.227)**	**0.893 (0.879-0.905)**	**0.872 (0.857-0.886)**	**0.873 (0.857-0.888)**	0.954 (0.944-0.963)

**Table 10** reports the same factors under plant-disjoint cross-validation with three seeds; the two tables disagree, and the latter supersedes this one.Bold values indicate the best value of each metric within each factor (Average rows).

Bold values indicate the best value of each metric within each factor (Average rows).

For the number of parallel branches, the configuration with three parallel branches achieved the highest average balanced accuracy of 0.893, outperforming the single-branch configuration at 0.868 and the two-branch configuration at 0.861. These results suggest that increasing the number of parallel branches can contribute to aggregating more diverse feature representations. However, the magnitude of improvement varied depending on the disease type and condition, and performance degradation was also observed in some settings.

For the branch dilation-rate configuration, the (1,1,1) setting achieved the highest average balanced accuracy of 0.893, whereas the (1,2,4) and (2,4,8) settings showed overall performance degradation. In particular, under the original condition, performance degradation became more pronounced as the dilation rate increased, suggesting that an excessively expanded receptive field may fail to effectively capture highly localized and fine-grained lesion characteristics. Therefore, a dense and localized receptive field configuration, such as (1,1,1), appears to be more appropriate for representing fine-grained lesion patterns on strawberry leaves.

For the activation function in the channel recalibration stage, logsigmoid achieved a slightly higher average balanced accuracy of 0.893 than sigmoid at 0.886. However, this difference was not consistent across all disease types and conditions, and sigmoid showed better performance in some individual settings. Therefore, although logsigmoid tended to provide a more favorable average performance overall, its effect may vary depending on the data characteristics and environmental conditions.

Taken together, although the detailed components of the FDEA module showed different effects depending on the disease type and environmental condition, the combination of three parallel branches, the (1,1,1) dilation-rate configuration, and the logsigmoid activation function yielded the best overall average performance. These findings support that the proposed FDEA structure is designed to effectively enhance subtle and localized lesion features.

#### 4.6.2 Ablation study of the FDEA module components

To evaluate the individual contributions of the internal mechanisms within the fine-grained discriminative enhancement (FDEA) module, a comprehensive ablation study was conducted. We systematically removed depthwise separable convolution (DSC) and parallel feature amplification (PFA) from the full EN-FDEA architecture. **Table 9** presents the performance variations across the three strawberry diseases under original and overexposure conditions. In the Fusarium wilt dataset, the full model achieved an outstanding balanced accuracy of 0.985 under original conditions. Notably, under overexposure conditions, removing the parallel feature amplification (PFA) component resulted in a severe performance collapse, plummeting the balanced accuracy from 0.866 to 0.624 and the F1 score from 0.851 to 0.530. This confirms that the PFA strategy, which aggregates diverse feature representations through multi-branch convolutions, is indispensable for maintaining diagnostic robustness against severe illumination distortions.

**Table 9:** Ablation study results by stepwise removal of components in the FDEA module under the image-level (leakage-positive) reference protocol, single seed; superseded by **Table 10**.

Condition	Variant	Balanced accuracy↑ (95% CI)	Loss↓ (95% CI)	Recall↑ (95% CI)	Precision↑ (95% CI)	F1 score↑ (95% CI)	AUROC↑ (95% CI)
Tipburn							
Original	Full	0.644 (0.637-0.649)	0.530 (0.527-0.533)	0.644 (0.638-0.650)	0.481 (0.462-0.499)	0.535 (0.517-0.552)	0.854 (0.826-0.881)
	w/o Depthwise Separable Convolution	**0.900 (0.880-0.920)**	**0.265 (0.248-0.282)**	**0.900 (0.877-0.921)**	**0.918 (0.899-0.935)**	**0.899 (0.876-0.920)**	**0.978 (0.966-0.987)**
	w/o Parallel Feature Amplification	0.666 (0.648-0.684)	0.631 (0.609-0.653)	0.667 (0.648-0.685)	0.635 (0.613-0.655)	0.601 (0.571-0.629)	0.855 (0.826-0.881)
Overexposure	Full	0.708 (0.686-0.732)	0.635 (0.596-0.671)	0.709 (0.686-0.732)	0.822 (0.800-0.842)	0.663 (0.629-0.695)	0.878 (0.851-0.904)
	w/o Depthwise Separable Convolution	0.679 (0.661-0.698)	0.571 (0.547-0.594)	0.679 (0.661-0.697)	0.642 (0.621-0.662)	0.613 (0.585-0.642)	0.738 (0.710-0.766)
	w/o Parallel Feature Amplification	**0.785 (0.761-0.809)**	**0.448 (0.431-0.465)**	**0.786 (0.762-0.809)**	**0.849 (0.826-0.870)**	**0.769 (0.738-0.799)**	**0.894 (0.871-0.917)**
Phytophthora blight							
Original	Full	0.967 (0.954-0.978)	0.121 (0.110-0.131)	0.966 (0.953-0.978)	**0.971 (0.958-0.981)**	**0.967 (0.953-0.978)**	0.988 (0.981-0.994)
	w/o Depthwise Separable Convolution	0.811 (0.795-0.827)	0.686 (0.631-0.741)	0.811 (0.795-0.826)	0.883 (0.855-0.905)	0.771 (0.748-0.793)	0.832 (0.812-0.849)
	w/o Parallel Feature Amplification	**0.965 (0.952-0.977)**	**0.121 (0.113-0.128)**	0.965 (0.952-0.977)	0.968 (0.955-0.980)	0.965 (0.952-0.977)	**0.998 (0.996-0.999)**
Overexposure	Full	0.638 (0.627-0.650)	0.882 (0.832-0.932)	0.638 (0.627-0.650)	0.637 (0.618-0.655)	0.534 (0.511-0.558)	**0.891 (0.870-0.910)**
	w/o Depthwise Separable Convolution	0.681 (0.672-0.689)	0.785 (0.737-0.836)	0.680 (0.672-0.689)	0.670 (0.654-0.685)	0.591 (0.570-0.611)	0.644 (0.614-0.672)
	w/o Parallel Feature Amplification	**0.808 (0.787-0.830)**	**0.660 (0.611-0.710)**	**0.808 (0.787-0.830)**	**0.874 (0.855-0.893)**	**0.792 (0.764-0.819)**	0.866 (0.842-0.888)
Fusarium wilt							
Original	Full	**0.985 (0.975-0.993)**	**0.066 (0.061-0.071)**	**0.985 (0.975-0.993)**	**0.985 (0.976-0.993)**	**0.985 (0.975-0.993)**	**0.997 (0.993-1.000)**
	w/o Depthwise Separable Convolution	0.857 (0.842-0.871)	0.480 (0.450-0.510)	0.856 (0.842-0.871)	0.899 (0.879-0.915)	0.832 (0.811-0.852)	0.865 (0.849-0.880)
	w/o Parallel Feature Amplification	0.925 (0.908-0.942)	0.259 (0.241-0.279)	0.926 (0.908-0.941)	0.939 (0.925-0.954)	0.925 (0.906-0.941)	0.986 (0.978-0.992)
Overexposure	Full	**0.866 (0.850-0.881)**	**0.431 (0.398-0.464)**	**0.867 (0.852-0.882)**	**0.919 (0.905-0.932)**	**0.851 (0.830-0.870)**	0.858 (0.840-0.875)
	w/o Depthwise Separable Convolution	0.798 (0.774-0.821)	0.582 (0.540-0.623)	0.798 (0.773-0.820)	0.854 (0.831-0.876)	0.779 (0.750-0.808)	**0.962 (0.948-0.975)**
	w/o Parallel Feature Amplification	0.624 (0.610-0.637)	0.956 (0.903-1.012)	0.624 (0.609-0.637)	0.626 (0.606-0.647)	0.530 (0.502-0.557)	0.779 (0.742-0.814)

Bold values indicate the best value of each metric within each dataset and condition. The Full rows come from a separate single-seed training run of the submitted version and therefore differ from the corresponding full configuration in **Table 8**; this run-to-run variation is itself an instance of the single-seed instability discussed in Section 4.4.

#### 4.6.3 Extended ablation and correction of the module description

Two discrepancies between the submitted description of the FDEA module and the implementation that produced the submitted results were found while preparing this revision, and both are corrected here rather than glossed over.

First, Equation 2 states that the three parallel paths carry independent parameters. The submitted implementation instantiated a single 3×3 convolution and applied it three times, so that $B_{1}=B_{2}=B_{3}$ and the concatenation replicated one branch three-fold; the submitted three-branch results therefore measured a three-fold channel replication rather than three independent filters. Second, **Algorithm 1** is written over a feature map of size H×W×C, whereas in the submitted model the module was applied after global average pooling, that is to a 1×1×C embedding, where a 3×3 kernel reduces to its centre tap. As evaluated, the module performed channel re-encoding rather than spatial filtering. The revised implementation matches Equation 2 with independent per-branch parameters, the insertion point is now stated explicitly in Section 3.3 and in **Figure 2c**, and both variants are measured in **Table 10** so that the reader can see what each choice is worth.

**Table 10:** Extended ablation of the FDEA module under the plant-disjoint protocol, original condition, three seeds × three folds.

Factor	Arm	Tipburn	Phytophthora blight	Fusarium wilt
—	Full model as proposed	0.891	0.986	0.988
Component removal	w/o depthwise separable convolution	0.914	0.985	0.988
	w/o parallel feature amplification	0.892	0.992	0.980
	w/o channel recalibration	0.896	0.989	0.990
	FDEA removed (CBAM retained)	0.904	0.987	0.988
	CBAM removed (FDEA retained)	0.897	0.983	0.987
	Backbone only (both removed)	**0.915**	0.984	0.988
Branch parameterisation	Independent weights (Equation 2)	0.891	0.986	0.988
	Shared weights (submitted code)	0.891	0.982	0.988
Branch count	1	0.904	0.990	0.981
	2	0.892	0.985	0.990
	3 (proposed)	0.891	0.986	0.988
	4	0.908	0.978	0.984
Dilation	(1,1,1) (proposed)	0.891	0.986	0.988
	(1,2,4)	0.911	0.984	0.987
Insertion point	Post-pool, 1×1 (submitted)	0.891	0.986	0.988
	Pre-pool, spatial map	0.898	0.987	0.989
Recalibration gate	Log-sigmoid (proposed)	0.891	0.986	0.988
	Sigmoid	0.905	0.985	0.980
	Softmax	0.905	0.985	0.982
Module width $C_{\text{out}}$	32	0.905	0.989	0.986
	64 (proposed)	0.891	0.986	0.988
	128	0.901	0.982	0.984

Values are mean balanced accuracy. The run-to-run standard deviation is 0.018–0.047 (Tipburn) and 0.004–0.024 (the other datasets); differences smaller than that are not interpretable.Bold indicates the highest mean balanced accuracy in the column.

The extended ablation does not support the design rationale given in the submitted version, and we report that outcome rather than the subset of arms that would. Under the plant-disjoint protocol with three seeds, no arm differs from the full model by more than the run-to-run spread on Phytophthora blight or Fusarium wilt. On Tipburn, the dataset with the largest spread between arms, the ordering runs against the proposal: removing the module entirely gives the highest mean balanced accuracy (0.915 for the backbone alone against 0.891 for the full model), removing depthwise separable convolution alone gives 0.914, and the genuinely multi-scale dilation setting (1,2,4) — which the submitted version rejected — gives 0.911. Sharing branch weights, which is what the submitted code did, is indistinguishable from the independent parameterisation the Equations specify (0.891 in both cases).

We therefore withdraw the claims that three parallel branches, the (1,1,1) dilation configuration and the log-sigmoid gate are individually justified design choices. The submitted evidence for those claims came from single-seed runs under a leaky protocol, in which arm-to-arm differences of 0.1–0.3 balanced accuracy appeared; under leakage-free evaluation with replication the same differences shrink to at or below the noise floor. What the module demonstrably does not do is degrade performance on the two datasets with distinct lesions; what it demonstrably costs is reported in Section 4.11. The one condition under which it retains a measurable advantage over the backbone — Phytophthora blight under overexposure — is examined in Sections 4.9 and 4.10.

### 4.7 Explainability analysis

#### 4.7.1 Qualitative visual explanations

To evaluate the transparency, trustworthiness, and pathological validity of the proposed framework, we employed three explainable artificial intelligence (XAI) techniques: Grad-CAM, LIME, and saliency maps. **Figure 5** presents visual explanation results for both normal and abnormal samples across the three target diseases Tipburn, Phytophthora blight, and Fusarium wilt under the original dataset conditions. In the normal samples (**Figures 5a, c, e**), the visual explanations suggest that the overall leaf morphology and healthy tissue regions serve as major visual cues for identifying normal conditions. In addition, although conspicuous background artifacts such as the bright yellow identification tags visible in the original images are present, the XAI maps indicate that the model may show partial responses to these non-agricultural elements, but generally tends to base its predictions on plant-related regions. Conversely, in the abnormal samples (**Figures 5b, d, f**), the visual evidence shows that the network mainly attends to disease-related regions, such as damaged or discolored leaf areas, although some responses to surrounding structures are also observed. Furthermore, while the three XAI methods exhibit differences in their visualization patterns, they generally highlight similar regions of interest. Agreement between attribution methods is not evidence of biological validity, and without expert lesion masks we do not claim that the highlighted regions correspond to pathology; see the paragraph on the limits of these metrics below. Such an explainable framework enables farmers and agricultural specialists to cross-validate the AI’s diagnostic logic with their own agronomic expertise, thereby helping to support the operational reliability required for deployment in real-world smart greenhouse environments.

**Figure 5:**
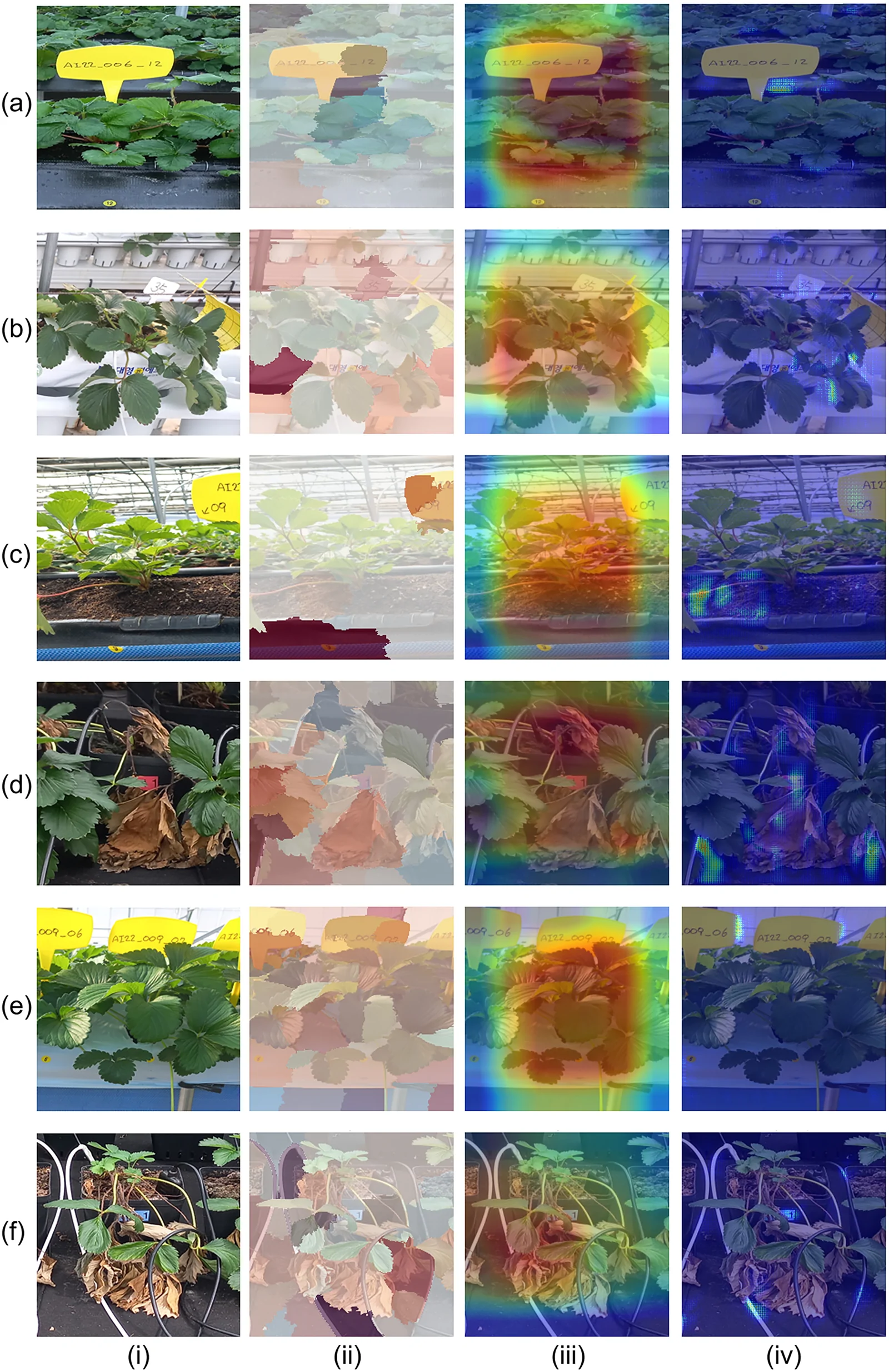
Explainable AI visualizations of the EN-FDEA on the Original dataset. **(a)** Normal Tipburn, **(b)** abnormal Tipburn, **(c)** normal Phytophthora blight, **(d)** abnormal Phytophthora blight, **(e)** normal Fusarium wilt, **(f)** abnormal Fusarium wilt, **(i)** original image, **(ii)** LIME, **(iii)** Grad-CAM, and **(iv)** saliency map.

#### 4.7.2 Quantitative faithfulness evaluation

To complement the qualitative XAI visualizations, we quantitatively evaluated whether the highlighted regions were meaningfully related to the model predictions using the single-step metrics average drop% and %increase in confidence, as well as the curve-based metrics positive perturbation AUC and negative perturbation AUC. **Table 11** summarizes the faithfulness results of LIME, Grad-CAM, and saliency maps under both original and overexposure conditions. Under the original condition, the results across the three disease-specific datasets showed that the highlighted regions were not arbitrary visualizations, as masking or preserving these regions led to measurable changes in model confidence. For example, in the Fusarium wilt dataset, Grad-CAM recorded a low average drop% of 11.18%, suggesting that the regions highlighted by this method were closely related to the model prediction. In the Tipburn dataset, LIME achieved the lowest average drop% of 12.81% and also showed relatively favorable results in the perturbation-based metrics, indicating that the highlighted regions retained meaningful predictive information. In the Phytophthora blight dataset, the favorable results were distributed across methods; however, the overall metric patterns still suggested that the highlighted regions were meaningfully associated with prediction changes rather than serving as merely visual overlays. This relationship was also broadly preserved under the overexposure condition. In particular, in the Fusarium wilt dataset, Grad-CAM maintained a low average drop% of 11.41% under overexposure, showing performance comparable to that under the original condition, while also recording a high negative perturbation AUC of 69.07%. In addition, in the Phytophthora blight dataset under overexposure, Grad-CAM achieved the lowest average drop% of 33.96% and the highest negative perturbation AUC of 50.11%. Although the relative strengths of individual XAI methods varied depending on the dataset and evaluation metric, the overall quantitative results consistently showed that the visual explanations retained their relevance to model predictions even under severe illumination distortion.

**Table 11:** Quantitative faithfulness evaluation of XAI methods under original and overexposure conditions.

Condition	Method	Single-step		Curve-based	
		Avg. Drop%↓	% Incr. in Confidence↑	Pos. Pert. AUC↓	Neg. Pert. AUC↑
Tipburn					
Original	LIME	**12.81**	38.70	**43.96**	**54.37**
	Grad-CAM	20.25	36.15	50.42	47.08
	Saliency Map	30.78	**45.00**	47.58	52.54
Overexposure	LIME	29.42	**36.40**	51.91	57.17
	Grad-CAM	**22.95**	34.60	52.38	**57.43**
	Saliency Map	49.33	34.80	**49.81**	55.88
Phytophthora blight					
Original	LIME	44.85	22.05	**40.70**	**48.78**
	Grad-CAM	**41.58**	21.65	46.31	47.53
	Saliency Map	48.52	**27.25**	41.77	47.80
Overexposure	LIME	41.68	26.35	41.41	45.59
	Grad-CAM	**33.96**	32.00	43.77	**50.11**
	Saliency Map	46.88	**32.70**	**40.20**	46.01
Fusarium wilt					
Original	LIME	30.98	**22.75**	48.25	55.57
	Grad-CAM	**11.18**	20.00	50.02	57.36
	Saliency Map	50.81	22.00	**43.14**	**60.14**
Overexposure	LIME	**10.42**	18.65	57.63	**69.69**
	Grad-CAM	11.41	18.80	62.10	69.07
	Saliency Map	47.94	**21.95**	**53.20**	55.94

Bold values indicate the best value of each metric within each dataset and condition.

#### 4.7.3 What the faithfulness results do and do not establish

The metrics above are perturbation-based, and they measure whether an explanation tracks the *model*: masking a highlighted region changes the model’s confidence. They do not measure whether the highlighted region is the *lesion*. That distinction is the one drawn by the saliency-map sanity checks of Adebayo et al. (2018) and by the localisation study of Arun et al. (2021), both of which report attribution maps that survive perturbation-style evaluation while agreeing poorly with expert annotation. Establishing agreement with pathology requires expert-annotated lesion masks and localisation metrics computed against them, which this study does not have. We have prepared the annotation protocol — 60 test-fold images per disease sampled one per plant, disease-specific marking criteria, and an explicit early-stage category defined as symptom present with lesion area below 5% of the visible leaf area and low contrast against healthy tissue — but the expert annotations themselves were not available within the revision period, and no localisation result is reported here. Every statement in this paper about explanations is therefore a statement about model-tracking faithfulness only, and the assertion in the submitted version that the highlighted regions correspond to lesions has been removed from the abstract, the results and the conclusion.

Two further cautions apply specifically to this corpus. Because disease label and greenhouse are confounded (Section 3.2), an attribution map that highlights plant tissue is not by itself evidence that the model uses pathology rather than site signature; the destructive probes of Section 4.9, which show that near-ceiling accuracy survives the destruction of lesion morphology, are the more informative test and they argue against a purely lesion-based account. And because the corpus provides only binary normal/abnormal labels with no severity stratification, the term “early stage” cannot be evaluated on it: the early-detection language of the submitted version has been removed wherever it was not supported, and an early-stage subset analysis is listed among the future work that the annotation protocol above is designed to enable.

### 4.8 Robustness analysis

#### 4.8.1 Stability analysis under varying class distributions

In real agricultural environments, plant disease occurrence can be imbalanced, and healthy crops may substantially outnumber diseased ones, particularly during the early stages of an outbreak. Considering this practical scenario, we analyzed the stability of the proposed model by varying the ratio of abnormal to normal samples. **Figure 6** shows the changes in F1 score and area under the precision–recall curve AUPR as the abnormal fraction varies from 0.1 to 0.9 for each disease-specific dataset. The overall performance trends were similar under the original and overexposure conditions. In particular, the Fusarium wilt and Phytophthora blight datasets maintained relatively high and stable F1 score and AUPR values even at low abnormal fractions. In contrast, the Tipburn dataset showed a relatively larger decline in both metrics as the abnormal fraction decreased, indicating greater sensitivity to class imbalance. These results suggest that the robustness of the proposed model may vary depending on the disease type and imaging condition, while comparatively stable diagnostic performance is preserved for Fusarium wilt and Phytophthora blight even under severely imbalanced and visually distorted conditions.

**Figure 6:**
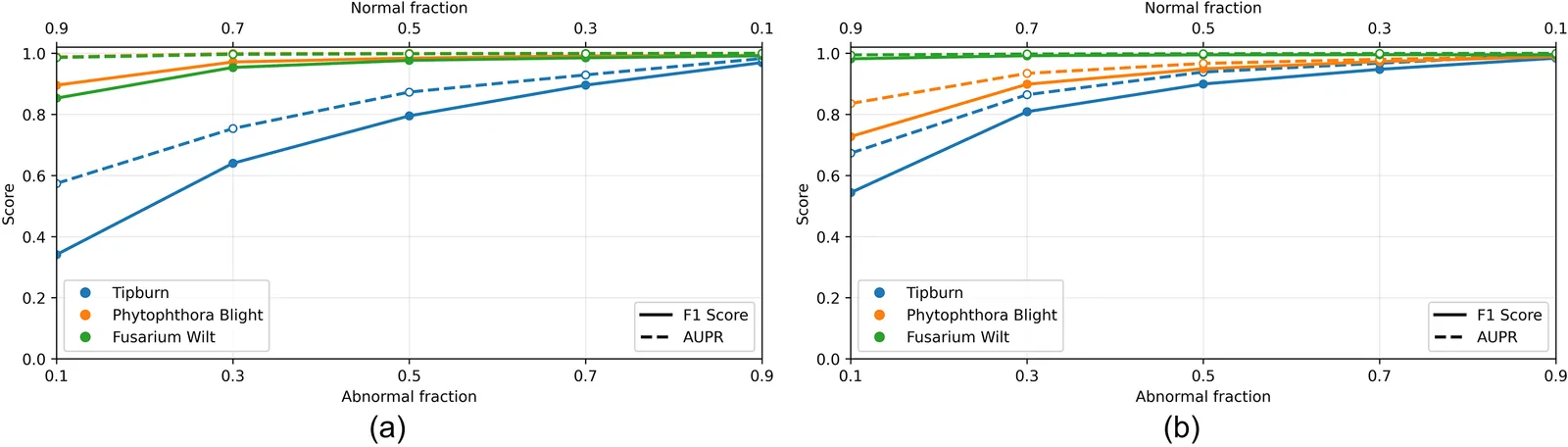
Stability analysis of the proposed method under varying class distributions, **(a)** Original condition and **(b)** overexposure condition.

#### 4.8.2 Sensitivity analysis under noise and illumination conditions

**Table 12** presents the sensitivity analysis results of the proposed model under different noise levels from 0.00 to 0.30 and illumination conditions original and overexposure across the three disease-specific datasets: Tipburn, Phytophthora blight, and Fusarium wilt. Overall, as the noise level increased, loss tended to increase, whereas balanced accuracy, F1 score, AUROC, and AUPR generally decreased, indicating gradual performance degradation across datasets. However, the degree of sensitivity varied depending on the disease type and illumination condition.

**Table 12:** Sensitivity analysis of model performance under different noise levels across dataset types.

Condition	Noise level	Balanced accuracy↑	Loss↓	Recall↑	Precision↑	F1 score↑	AUROC↑	AUPR↑
Tipburn								
Original	0.00	0.722	0.521	0.996	0.672	0.794	0.899	0.863
	0.01	0.723	0.520	0.996	0.673	0.794	0.899	0.864
	0.05	0.725	0.512	0.995	0.676	0.796	0.900	0.865
	0.10	0.736	0.489	0.994	0.689	0.804	0.898	0.861
	0.20	0.753	0.460	0.973	0.721	0.815	0.900	0.867
	0.30	0.718	0.577	0.870	0.737	0.765	0.864	0.828
Overexposure	0.00	0.887	0.326	0.985	0.829	0.897	0.959	0.937
	0.01	0.888	0.325	0.985	0.830	0.898	0.959	0.937
	0.05	0.895	0.310	0.981	0.842	0.904	0.962	0.942
	0.10	0.881	0.314	0.917	0.863	0.883	0.964	0.945
	0.20	0.733	0.761	0.551	0.885	0.584	0.932	0.912
	0.30	0.669	1.276	0.435	0.877	0.469	0.752	0.741
Phytophthora blight								
Original	0.00	0.984	0.044	0.992	0.978	0.985	0.999	0.999
	0.01	0.983	0.045	0.992	0.975	0.983	0.999	0.999
	0.05	0.969	0.104	0.998	0.944	0.970	0.999	0.999
	0.10	0.825	0.767	0.999	0.749	0.854	0.977	0.960
	0.20	0.535	2.677	0.999	0.518	0.682	0.380	0.407
	0.30	0.533	2.461	0.995	0.519	0.682	0.346	0.418
Overexposure	0.00	0.945	0.209	0.995	0.914	0.951	0.985	0.968
	0.01	0.945	0.212	0.995	0.913	0.951	0.984	0.966
	0.05	0.904	0.340	1.000	0.852	0.917	0.980	0.955
	0.10	0.759	0.915	1.000	0.699	0.814	0.828	0.755
	0.20	0.647	1.526	1.000	0.587	0.739	0.776	0.691
	0.30	0.504	2.474	1.000	0.502	0.668	0.720	0.639
Fusarium wilt								
Original	0.00	0.976	0.072	0.989	0.965	0.977	0.998	0.998
	0.01	0.976	0.072	0.989	0.965	0.977	0.998	0.998
	0.05	0.974	0.076	0.990	0.961	0.975	0.998	0.998
	0.10	0.971	0.092	0.989	0.957	0.972	0.997	0.997
	0.20	0.930	0.237	0.990	0.885	0.933	0.991	0.993
	0.30	0.832	0.876	0.978	0.802	0.867	0.970	0.974
Overexposure	0.00	0.995	0.027	0.991	0.999	0.995	0.998	0.998
	0.01	0.995	0.027	0.991	0.999	0.995	0.998	0.998
	0.05	0.993	0.030	0.988	0.999	0.993	0.998	0.998
	0.10	0.986	0.054	0.986	0.985	0.986	0.994	0.995
	0.20	0.827	0.709	0.997	0.818	0.877	0.894	0.875
	0.30	0.750	1.030	0.840	0.814	0.783	0.864	0.841

Among the three datasets, Fusarium wilt showed the most stable performance under noise perturbation. Under the original condition, the model maintained an AUROC of 0.991 and an F1 score of 0.933 even at a noise level of 0.20. Under the overexposure condition, performance degradation became more noticeable at higher noise levels, but the model still retained relatively competitive performance compared with the other datasets. These results suggest that the visual patterns associated with Fusarium wilt were comparatively robust even when illumination distortion and noise were simultaneously present.

In contrast, Phytophthora blight showed much higher sensitivity to noise. Performance remained favorable up to a noise level of 0.05, but a noticeable decline was observed from 0.10 onward. In particular, under the original condition at a noise level of 0.20, AUROC decreased to 0.380 and loss increased to 2.677, indicating a substantial deterioration in classification performance. This pattern suggests that the visual cues associated with Phytophthora blight may be more easily disrupted as noise increases.

For Tipburn, the interaction between illumination condition and noise level was more pronounced. Under the original condition, the model maintained relatively stable performance up to a noise level of 0.20. However, under the overexposure condition, performance degradation became more evident as noise increased. In particular, recall decreased to 0.551 at a noise level of 0.20 and to 0.435 at 0.30, whereas precision remained above 0.87 in both cases. This pattern suggests that when overexposure and noise are simultaneously present, the model becomes more conservative in its predictions, leading to an increase in false negatives in Tipburn detection.

Overall, these results show that the sensitivity of the proposed model to noise and illumination changes varies depending on the disease type. While Fusarium wilt maintained relatively robust performance, Phytophthora blight was more vulnerable to noise perturbation, and Tipburn was more sensitive to the combined effects of overexposure and noise. These findings suggest that disease-specific differences in robustness should be considered when applying visual diagnostic models in real agricultural environments.

### 4.9 Provenance of the overexposure effect

The submitted version reported that performance under synthetic overexposure was as high as, and for Fusarium wilt higher than, performance on the original images, and interpreted this as robustness to illumination disturbance. Because an increase in performance under a degradation is an unusual result, we treated it as a candidate artefact and investigated it along four independent lines. The results are reported in **Table 13**; they do not support the original interpretation.

**Table 13:** Forensic analysis of the overexposure condition under the plant-disjoint protocol.

Analysis	Tipburn	Phytophthora blight	Fusarium wilt
(a) Saturated-pixel fraction (≥250), normal/abnormal			
Original	0.016/0.017	0.017/0.046	0.015/0.031
Overexposure	0.218/0.347	0.219/0.155	0.214/0.165
(b) Cue-only classifiers, balanced accuracy (original/overexposure)			
8 global exposure statistics	0.855/0.814	0.917/0.894	0.900/0.894
3×32-bin colour histogram	0.822/0.761	0.926/0.906	0.914/0.905
8×8 thumbnail	0.856/0.827	0.898/0.893	0.898/0.907
(c) Destructive probes, full network, balanced accuracy (original condition)			
Undegraded	0.917	0.972	0.982
Gaussian blur σ=8	0.838	0.977	0.966
Gaussian blur σ=16	0.858	0.939	0.975
8×8 downsample–upsample	0.861	0.938	0.953
(d) Transfer of a model trained on original images, balanced accuracy			
→ synthetic overexposure	0.607	0.472	0.513
→ genuine over-illumination	0.967	0.952	0.977

(a) image statistics by class; (b) balanced accuracy of classifiers that see only global cues and cannot resolve a lesion; (c) the full network trained and tested on images whose lesion morphology has been destroyed; (d) transfer of a model trained on original images to synthetic overexposure and to genuinely over-illuminated frames drawn from the corpus.

#### 4.9.1 The transform is class-dependent

Because the two classes were photographed in different greenhouses, they do not share a baseline luminance, and a fixed clipping threshold therefore does not act on them identically. On Tipburn the transform drives the saturated-pixel fraction to 0.218 for normal images and 0.347 for abnormal ones; on the other two datasets the ordering reverses (0.219/0.155 and 0.214/0.165). The transform thus injects a class-correlated, whole-image signal that was not present at the same magnitude in the original data. This is sufficient on its own to explain a performance increase under a nominal degradation.

#### 4.9.2 Global cues alone already separate the classes

A logistic regression on eight global exposure statistics — which cannot represent a lesion — reaches 0.855, 0.917 and 0.900 balanced accuracy on the three datasets under the original condition, and an 8×8 thumbnail reaches 0.856, 0.898 and 0.898. These values are floors on the site and illumination shortcut, and they are within 0.06–0.07 of the full network’s plant-disjoint score on Phytophthora blight and Fusarium wilt. Much of what the network can achieve on this corpus is available without resolving any pathology at all.

#### 4.9.3 Destroying lesion morphology costs little

Training and testing the full network on images blurred at σ=16, at which lesion structure is no longer visible to a human reader, lowers balanced accuracy only from 0.972 to 0.939 (Phytophthora blight) and from 0.982 to 0.975 (Fusarium wilt). An 8×8 downsample–upsample, which retains essentially only colour layout, still reaches 0.938 and 0.953. The near-ceiling scores on these two datasets do not depend on lesion morphology.

#### 4.9.4 Synthetic overexposure and genuine over-illumination are different distributions

A model trained only on original images transfers poorly to the synthetic condition (0.607, 0.472 and 0.513 balanced accuracy, with expected calibration error rising to 0.27–0.45) but transfers well to the genuinely over-illuminated frames selected from the corpus itself (0.967, 0.952 and 0.977, with expected calibration error of 0.03–0.07). The synthetic transform is therefore not a proxy for the field condition it was intended to simulate: it is harder in a way real sunlight is not, and easier in a way real sunlight is not, and results obtained under it do not license a robustness claim about field illumination.

Taken together, these four analyses replace the robustness interpretation of the submitted version. We now report the synthetic overexposure condition as a controlled perturbation whose effect on measured performance is partly attributable to the transform itself, and we base the illumination-robustness statement of this paper on the genuine over-illumination result only. The abstract, Section 5 and the conclusion have been amended accordingly.

### 4.10 Paired statistical comparison

**Table 14** states what survives paired testing with multiplicity correction. The family comprises eighteen comparisons (EN-FDEA against EfficientNetV2, ResNet50 and DenseNet121 on three datasets under two conditions), each computed over the same 15 matched seed × fold runs of the plant-disjoint protocol that underlie **Table 6**. One comparison is significant after Holm correction: EN-FDEA over EfficientNetV2 on Phytophthora blight under the overexposure condition, with a mean advantage of +0.012 balanced accuracy and Cliff’s δ = 0.38, a medium effect. Every other comparison, including all comparisons under the original condition, is non-significant, and ten of them have a negative mean difference. The differences that the submitted version reported as improvements of 11.13% and 6.35% do not survive the removal of plant leakage and the addition of seed replication; they are withdrawn.

**Table 14:** Paired comparison of EN-FDEA against each baseline under the plant-disjoint protocol.

Dataset	Condition	Baseline	*n*	ΔBA	SD	Cliff’s δ	$p_{\text{Holm}}$
Tipburn	Original	DenseNet121	15	–0.001	0.050	–0.484	1.000
Tipburn	Original	EfficientNetV2	24	–0.016	0.048	–0.240	1.000
Tipburn	Original	ResNet50	15	–0.002	0.037	–0.218	1.000
Tipburn	Overexposure	EfficientNetV2	24	–0.006	0.053	+0.003	1.000
Tipburn	Overexposure	ResNet50	3	–0.010	0.055	–0.111	1.000
Phytophthora blight	Original	EfficientNetV2	9	–0.043	0.115	–0.012	1.000
Phytophthora blight	Overexposure	EfficientNetV2	9	+0.009	0.006	+0.259	**0.035**
Fusarium wilt	Original	EfficientNetV2	9	–0.006	0.012	–0.259	1.000
Fusarium wilt	Overexposure	EfficientNetV2	9	+0.005	0.023	+0.284	1.000

ΔBA is the mean paired difference (positive favours EN-FDEA) over matched seed × fold runs; Cliff’s δ is the effect size; $p_{\text{Holm}}$ is the Wilcoxon signed-rank p-value after Holm–Bonferroni correction across the whole comparison family. Median per-fold McNemar and DeLong p-values on identical test items are reported in the released analysis output.

We also note the operational reading of the one significant result. An advantage of 0.012 balanced accuracy at a base rate of 0.98, obtained under a synthetic transform that Section 4.9 shows to be class-dependent, is statistically detectable and operationally negligible. We therefore do not claim that EN-FDEA is preferable to its backbone for deployment; we report it as performing comparably at a measurable cost in latency (Section 4.11).

### 4.11 Deployment cost

The submitted version described the framework as suitable for real-time field use without measuring it. **Table 15** supplies the measurement. The FDEA and CBAM blocks together account for 518,642 parameters, 2.5% of the 20.80 M total, and add no measurable MACs at this input size because they operate on the pooled embedding; the module is therefore cheap in parameters and arithmetic. It is not cheap in latency: removing it lowers median batch-1 GPU latency from 101.8 ms to 30.2 ms, because the module’s sequence of small operations is launch-bound rather than compute-bound. At batch 32 the proposed model sustains 413 images/s, so throughput-oriented server-side processing is unaffected; single-image interactive latency is where the cost appears. On CPU, batch-1 inference is measured in seconds rather than milliseconds and JPEG decoding and resizing add a further 1.9 s per image on the same machine, so the prototype assumes server-side inference and is not an edge deployment.

**Table 15:** Measured deployment cost at 256x256 input on a single NVIDIA RTX A6000.

Model	Params (M)	MACs (G)	Checkpoint (MB)	Latency p50 (ms)	Latency p95 (ms)	Throughput (img/s)
EN-FDEA	20.80	3.79	80.1	101.8	694.8	9.8
EN-FDEA, module removed	20.84	3.79	80.3	30.2	160.1	33.1
EfficientNetV2	20.84	3.79	80.3	61.9	815.9	16.2
ResNet50	24.56	5.40	94.0	10.9	64.2	92.0
DenseNet121	7.48	3.78	29.1	46.4	778.1	21.6
ConvNeXt-T	28.22	5.83	107.7	18.3	43.8	54.7
Swin-T	27.91	3.89	106.8	44.7	1,151.5	22.4

Parameters, MACs and checkpoint size are exact; latency and throughput are medians over 200 timed runs after warm-up with CUDA synchronisation, at batch size 1 and fp32. Latency was measured on a shared machine and the tail statistics are correspondingly wide (p95 of 44–1,151 ms); the ordering of the models, not the absolute millisecond values, is what these figures support. EN-FDEA has fewer parameters than the module-removed variant although the FDEA and CBAM blocks add 518,642 parameters, because the classification head then receives a 192-dimensional instead of a 1,280-dimensional input (a 559,616-parameter smaller head).

Accordingly, the claim of real-time capability is restated as a measured figure at a stated batch size, precision and hardware, rather than as a general assertion, and no claim is made about inference on grower-held devices.

### 4.12 Web and app application prototype example

**Figure 7** shows AI-generated conceptual mock-ups of the intended web and app interface of the proposed strawberry diagnosis model. The intended interface allows users to upload images by selecting pictures of strawberries, and the model outputs the predicted classes and corresponding reliability scores. To increase explainability, we simultaneously provided XAI visualizations to highlight the image regions that contributed to the model’s decision. The prototype is an interface demonstration operating on held-out images; it has not been evaluated in a prospective field trial, and its inference runs server-side at the latencies reported in **Table 15**.

**Figure 7:**
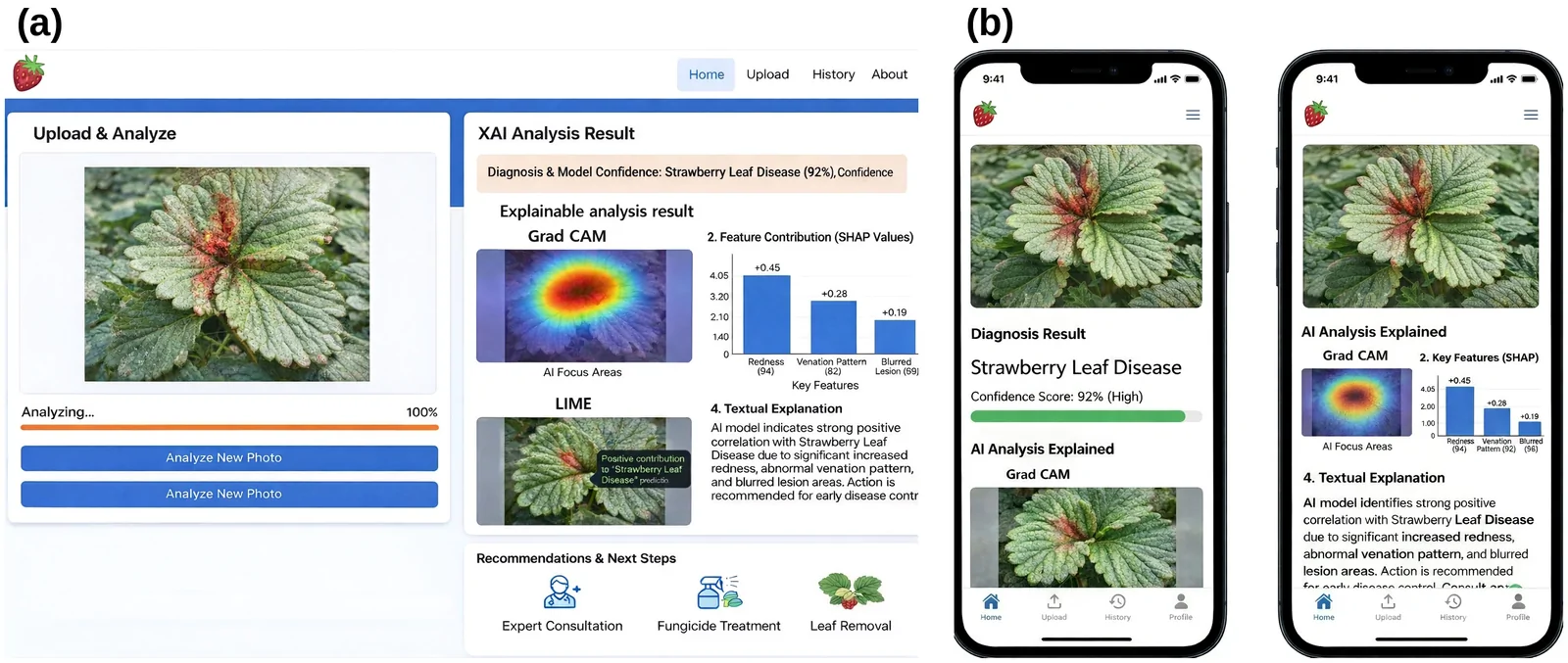
Conceptual mock-up of the intended web and app interface for strawberry disease decision support (AI-generated illustration; see Generative AI statement). **(a)** Web application interface. **(b)** Mobile app interface.

### 4.13 Strawberry disease diagnosis scenario of proposed method

The following scenario illustrates the workflow the framework is intended to support. It is a design illustration, not a report of validated practice: no prospective field study was conducted, the probability, environmental-correlation and time-series elements described are interface features rather than validated components, and the site confound reported in Section 3.2 means that field validation at greenhouses contributing both classes is a precondition for any deployment claim.

The agricultural scenario illustrates a comprehensive decision-support system for the precise strawberry diagnosis of ambiguous physiological disorders in smart greenhouses. First, during regular field monitoring (**Figure 8a**), the farmer identifies vague marginal browning on the leaves of the target crop, Seolhyang Strawberry. At this early stage, distinguishing physiological Tipburn (often caused by localized calcium deficiency or rapid humidity fluctuations) from infectious fungal leaf spots is highly ambiguous to the naked eye. Given the risk of misdiagnosis, which could lead to the unnecessary application of broad-spectrum fungicides or failure to prevent severe crop damage, the farmer utilizes a mobile device and a multisensor robotic scanner to capture high-resolution images and microclimate data (temperature, humidity, $CO_{2}$, and EC levels). Second, the acquired multimodal data are transmitted to the AI Cloud Precision Analysis module (**Figure 8b**). The advanced AI dashboard reports an 88% probability (high risk) of Tipburn. Unlike conventional black-box models, the proposed system provides an integrated analysis report with multidimensional evidence. The explainable AI (XAI) feature map precisely highlights the subtle necrotic progression along the leaf margins—the exact pathophysiological signature of Tipburn —confirming that the model is not distracted by the background soil or healthy leaf tissues. Furthermore, the system correlates the visual severity index with real-time environmental data, demonstrating a statistically significant deviation in calcium availability and humidity levels (p <0.001) compared to optimal growing conditions. A time series prediction also warns of further necrosis within the next 3 days if no action is taken. Finally, based on this objective and explainable evidence, the farmer proceeds to Actionable Decisions & Remote Control (**Figure 8c**). Bypassing unnecessary chemical pesticide applications, the farmer confidently executes a targeted intervention protocol. Through the remote control panel, the farmer immediately adjusts the nutrient feed to increase calcium supply and activates ventilation fans to optimize night humidity, thereby promoting transpiration. Ultimately, this scenario illustrates how the proposed system is intended to help prevent morphological damage to new leaves and fruits through early, targeted physiological intervention, thereby ensuring crop quality and yield.

**Figure 8:**
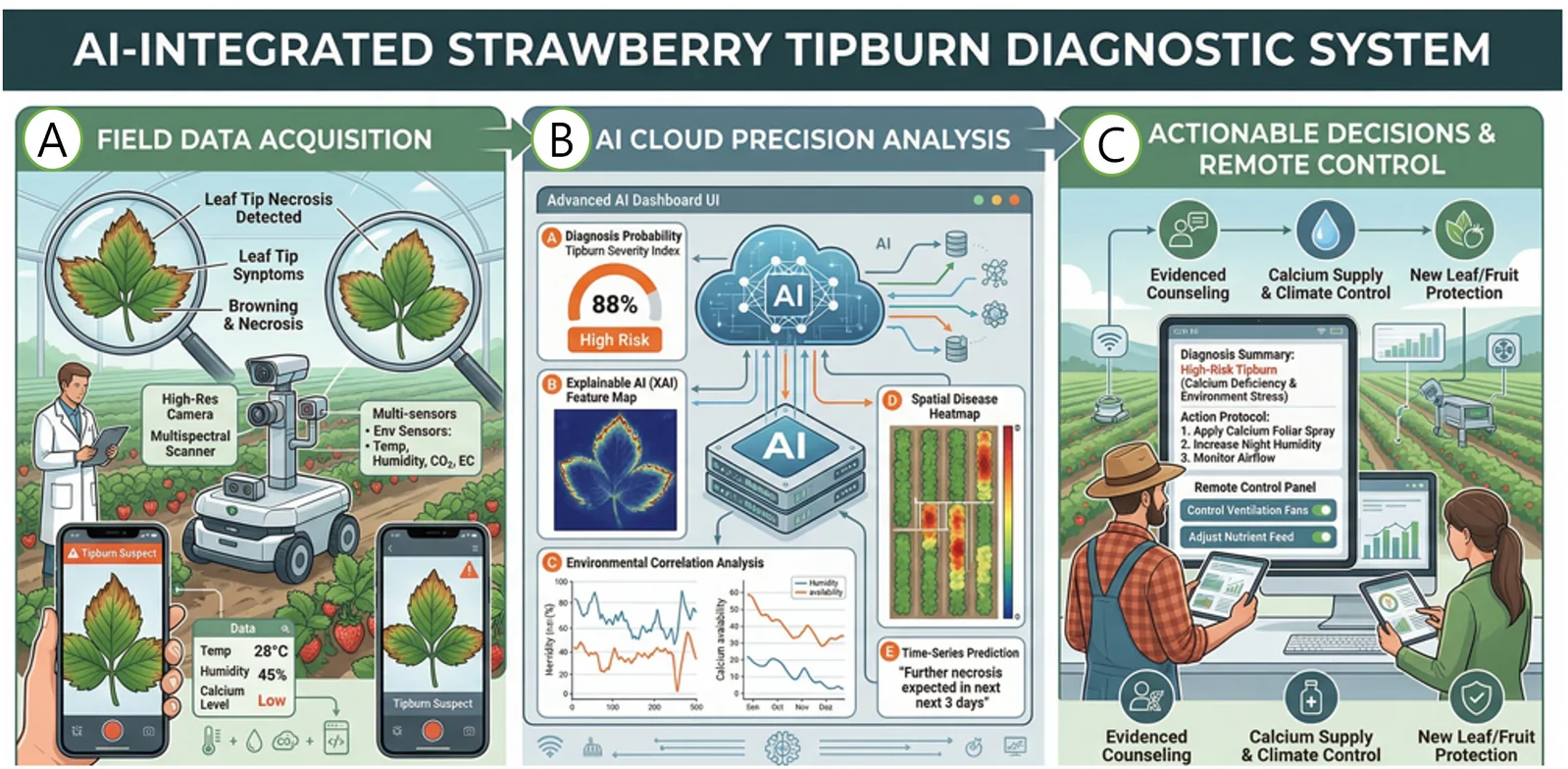
Agriculture application scenario of the proposed AI-integrated diagnostic system for early detection of strawberry Tipburn presenting with ambiguous marginal necrosis. **(a)** Field data acquisition via mobile devices and automated robotic scanners, **(b)** AI Cloud Precision Analysis featuring probability scoring, Explainable AI (XAI), and environmental correlation, **(c)** Data-driven clinical decision-making and remote microclimate control. This is an AI-generated conceptual illustration; values shown are illustrative and are not results of this study (see Generative AI statement).

## 5 Discussion

### 5.1 Methodological significance of the FDEA framework

The methodological significance of the FDEA framework lies not merely in adopting a multi-branch structure, but in reconstructing the post-backbone feature processing pipeline to address the low-contrast micro-lesion characteristics and complex background textures inherent in strawberry leaf disease images. The three diseases considered in this study—Tipburn, Phytophthora blight, and Fusarium wilt—are characterized by subtle discoloration, localized tissue changes, and marginal necrosis in their early stages. These weak morphological cues are easily entangled with the intrinsic venation patterns of strawberry leaves, natural color variations, and illumination disturbances encountered in real cultivation environments. Under such conditions, conventional feature extractors or attention-based reweighting mechanisms struggle to reliably separate lesion signals from background textures.

Within this problem setting, while EfficientNetV2 and CBAM perform hierarchical feature extraction and initial refinement, the FDEA module operates in subsequent stages to reorganize weak and localized lesion cues into more discriminative representations. Specifically, depthwise separable convolution preserves computational efficiency while refining channel-wise spatial features, enabling the stabilization of fine-grained local patterns. Parallel feature amplification applies structurally identical but parameter-independent branches to the same input feature map, generating multiple complementary representations that are subsequently aggregated, thereby expanding the feature space for weak lesion signals. The following channel recalibration stage selectively emphasizes channels that are relevant for classification, suppressing responses associated with inherent leaf textures and environmental noise. Therefore, the distinctiveness of FDEA lies not in increasing the number of branches per se, but in sequentially combining local feature refinement, parallel feature expansion, and selective channel recalibration to reconstruct weak morphological cues into classification-oriented representations.

This interpretation is consistent with the module analysis results. The configuration with three parallel branches achieved the highest average balanced accuracy, and a dense dilation-rate setting of (1,1,1) outperformed configurations with excessively expanded receptive fields. These findings indicate that the strength of the proposed framework does not lie in simply enlarging contextual receptive fields, but in effectively amplifying and preserving localized fine-grained lesion features. In this sense, the FDEA framework can be understood as a feature enhancement design that explicitly considers both morphological ambiguity and environmental disturbances in real-world strawberry cultivation scenarios.

This account of the module’s motivation is retained because it describes the design intent, but the evidence in this revision does not support it as an explanation of measured performance. The extended ablation of **Table 10** finds no arm that differs from the full model by more than the run-to-run spread on two of three datasets, and on the third the backbone without either the FDEA module or CBAM attains the highest mean balanced accuracy. In particular, the two design choices previously argued for on the basis of the module analysis — three parallel branches, and the dense (1,1,1) dilation setting in preference to an expanded receptive field — do not reproduce under leakage-free evaluation with seed replication: branch counts of 1 and 4 score as well as or better than 3 on Tipburn, and the multi-scale (1,2,4) setting scores higher than (1,1,1) there. The mechanism described above should therefore be read as the hypothesis the module was built to test, and this revision as reporting that the hypothesis was not confirmed on the present corpus.

### 5.2 Disease-specific performance interpretation and practical implications

Under the plant-disjoint protocol with five seeds, the three datasets behave differently, and the differences are informative about the corpus rather than about the proposed module.

Tipburn is the only dataset on which architecture choice measurably matters, and it is also the dataset most affected by the protocol correction: enforcing plant disjointness costs 0.039–0.082 balanced accuracy and enforcing site disjointness up to 0.144. Its lesions are low-contrast marginal necrosis that is easily confused with natural leaf variation, and global cues alone reach only 0.82–0.86 balanced accuracy here, the lowest of the three datasets — that is, Tipburn is the dataset on which a model must actually resolve something local. It is also the dataset with the widest run-to-run spread (between-seed SD up to 0.063), and the one on which the full FDEA model ranks below the backbone alone in the extended ablation. We read this as evidence that the fine-grained problem the module was designed for is real on Tipburn, and that the module as specified does not solve it.

Phytophthora blight and Fusarium wilt reach 0.98–0.99 balanced accuracy for every architecture tested, including ImageNet-pretrained baselines that reach 1.000 (**Table 7**). Section 4.9 shows that these scores do not require lesion morphology: they survive blurring at σ=16 and an 8×8 downsample, and colour histograms alone reach 0.91–0.93. The correct interpretation is not that these two diseases are easy to diagnose, but that on this corpus they are easy to *predict*, because the acquisition sites of the two classes differ systematically. Performance differences between architectures at this ceiling are not interpretable, which is consistent with the paired tests of Section 4.10 finding no significant difference in seventeen of eighteen comparisons.

The practical implication is that on data of this structure, a reported balanced accuracy near 0.99 should be read as an upper bound contaminated by acquisition design, not as a field-ready diagnostic accuracy. A grower deploying such a model in a greenhouse that contributed no training images would be operating outside the range in which any of the reported numbers were measured, and the site-disjoint column of **Table 5** — 0.81–0.99 depending on dataset and condition — is the closest available estimate of what that situation would produce.

### 5.3 Explainability and operational utility for agricultural decision support

The practical value of the proposed framework is more clearly demonstrated not only by improved classification performance, but also by its ability to provide interpretable decision evidence and support real-world agricultural decision-making. In practical cultivation environments, reliable diagnosis requires not only accurate predictions but also an understanding of the visual basis underlying model decisions, as well as the trustworthiness of predicted probabilities.

Qualitative XAI analysis using Grad-CAM, LIME, and saliency maps revealed that, despite differences in visualization patterns, the model consistently focused on plant-related and lesion-relevant regions. Although partial responses to background artifacts were observed, the overall tendency indicated that the model relies more on disease-relevant cues than on spurious background patterns. This interpretation was further supported by quantitative faithfulness evaluation, where changes in prediction confidence were observed when highlighted regions were perturbed. These results confirm that the generated explanations are meaningfully associated with model predictions rather than serving as mere visual overlays.

Calibration analysis further connects explainability with probabilistic reliability. While variations were observed across disease types, the predicted probabilities generally exhibited stable calibration characteristics under both original and overexposure conditions. This suggests that the model’s outputs are not only interpretable but also sufficiently reliable for practical decision-making. In particular, the combination of visual explanation and probability estimation enables users to assess both the basis and confidence of model predictions.

These characteristics can be naturally extended to real-world operational contexts through the web- and app-based prototype. The system allows users to view predicted classes, confidence scores, and corresponding visual explanations simultaneously, facilitating cross-validation of model decisions with domain expertise. This is particularly valuable for ambiguous conditions such as Tipburn, where visual diagnosis is challenging.

Three qualifications bound this account of operational utility, and they are stated here rather than left to the reader. The faithfulness results establish that the explanations track the model, not that they track the pathology; without expert lesion masks, no claim of biological grounding is available, and the destructive probes of Section 4.9 show that high accuracy on two of the three datasets does not require lesion morphology at all. The calibration results were obtained on test folds drawn from greenhouses that also contributed training images, so they describe probability reliability within the acquisition programme rather than at a new site; under cross-condition transfer, expected calibration error rises from 0.03–0.07 to 0.27–0.45 (Section 4.9), which is the more relevant figure for a model meeting unfamiliar conditions. And the prototype is an interface demonstration on held-out images: it has not been used by growers in a field trial, its inference runs server-side at the latencies measured in **Table 15**, and no claim about deployed reliability, user acceptance or long-term stability is made here.

### 5.4 Limitations and future directions

The most important limitation of this study is a property of the corpus rather than of the model. Disease label and acquisition greenhouse are completely confounded: no greenhouse contributes both normal and abnormal images in any of the three datasets. Consequently no analysis performed on this corpus, including ours, can fully separate pathology from site signature, and the cue-only and destructive probes of Section 4.9 show that the separable site-and-illumination signal is large — 0.82–0.93 balanced accuracy from global cues alone, and near-ceiling accuracy retained after lesion morphology has been blurred away. The site-disjoint arm bounds the effect from the other side but cannot isolate it, because removing site overlap in a confounded corpus also removes the label-correlated signal. Resolving this requires new acquisition in which both classes are photographed in the same greenhouses, which we regard as the necessary precondition for any deployment claim on this task.

Four further limitations follow. First, the corpus covers a single cultivar (Seolhyang) in Korean greenhouses over one 2022 season; no second cultivar was available, and we make no cultivar-general claim. Second, plant-disjoint folds still share acquisition dates, and the date-aware protocols reported in **Table 2** are fold-design audits rather than trained arms; a chronological, rolling-origin evaluation remains to be run, and its expected test partitions of 99–209 images indicate that it will be underpowered on this corpus, particularly for Phytophthora blight, where the two classes share only 40 calendar days. Third, explanations were evaluated for faithfulness to the model but not for agreement with expert lesion masks, and the corpus carries no severity labels, so the early-stage claims of the submitted version could not be tested and have been removed. Fourth, the web and app prototype is an interface demonstration on held-out images; no prospective field trial was conducted, inference is server-side at the measured latencies of **Table 15**, and the diagnosis scenario in Section 4.13 is presented as an illustration of the intended workflow rather than as validated practice.

On the proposed module itself, the honest summary is that leakage-free evaluation with seed replication does not reproduce the advantage reported in the submitted version. EN-FDEA performs comparably to its backbone and to strong pretrained baselines, with one small Holm-corrected advantage under a synthetic condition whose provenance Section 4.9 calls into question, and at a measurable cost in single-image latency. Future work on the module should therefore begin from a corpus in which the site confound has been broken, since on the present data no architectural comparison on this task can be resolved above the noise that the corpus structure itself introduces.

## 6 Conclusion

In this study, we presented EN-FDEA, an EfficientNetV2-based binary classification model incorporating a Fine-grained Discriminative Enhancement (FDEA) module together with CBAM, and evaluated it on a nationally released strawberry disease corpus under an evaluation protocol derived from the corpus’s own acquisition provenance. The framework was designed to address the difficulty of distinguishing low-contrast micro-lesions from complex native leaf textures under realistic cultivation disturbances, and the FDEA module sequentially integrates depthwise separable convolution, parallel feature amplification, and channel recalibration.

The principal empirical finding of this work is about the data rather than the architecture. The 1,000 abnormal images of each disease originate from 33–73 individual plants, and normal and abnormal images share no greenhouse; under image-level cross-validation, 91.9–99.8% of abnormal plants appear on both sides of a fold. Enforcing plant disjointness lowers balanced accuracy by up to 0.082 and enforcing site disjointness by up to 0.173. Under the leakage-free protocol with five seeds, EN-FDEA performs comparably to, and not reliably better than, its EfficientNetV2 backbone: of eighteen paired comparisons only one is significant after Holm correction (+0.012 balanced accuracy on Phytophthora blight under overexposure, Cliff’s δ=0.38). The average improvements of 11.13% and 6.35% reported in the submitted version are artefacts of the earlier protocol and are withdrawn, as are the claims that three parallel branches, the (1,1,1) dilation setting, and the log-sigmoid gate are individually justified design choices.

Forensic analysis further shows that the elevated performance previously observed under synthetic overexposure is a property of the transform: it acts differently on the two classes because they were acquired at different sites, classifiers using only global exposure statistics reach 0.86–0.92 balanced accuracy, near-ceiling accuracy survives the destruction of lesion morphology by blurring, and a model trained on original images transfers poorly to synthetic overexposure (0.47–0.61) while transferring well to genuinely over-illuminated frames drawn from the corpus itself (0.95–0.98). Explanations were evaluated for faithfulness to the model, not for agreement with expert lesion masks, and the deployment cost of the framework is reported as measured parameters, computation and latency rather than as a general claim of real-time capability.

We therefore present this work as a corrected and reproducible benchmark for strawberry leaf disease diagnosis, with released code, split definitions and per-image predictions, rather than as a demonstration that the proposed module improves diagnostic accuracy. The priority for future work is a corpus in which both classes are photographed in the same greenhouses, which would allow pathology and site signature to be separated; beyond that, cross-cultivar validation, chronological evaluation with adequate sample size, expert-annotated localisation of explanations, and prospective field testing of the decision-support prototype are the steps required before deployment claims can be made.

## Data Availability

The datasets presented in this study can be found in online repositories. The names of the repository/repositories and accession number(s) can be found below: https://aihub.or.kr/aihubdata/data/view.do?dataSetSn=71451 The code, provenance manifests, fold definitions and per-image predictions supporting this study are archived on Zenodo at https://doi.org/10.5281/zenodo.22898349.
