# Supplementary material for: Explainable fine-grained feature enhancement framework for strawberry diagnostic decision making
Source: Front Plant Sci. 2026 Oct 9;17:1941698. doi: 10.3389/fpls.2026.1941698 (PMC13690960; doi:10.3389/fpls.2026.1941698)
Supplement: Supplementary file 1 [file DataSheet1.docx]

# Supplementary Material: Explainable Fine-grained Feature Enhancement Framework for Strawberry Diagnostic Decision Making

# S1. Methodology

## S1.1. Evaluation Metrics Definition

We provide the formal definitions and mathematical expressions of the evaluation metrics used in the main manuscript. The confusion-matrix terms are denoted as true positives (TP), true negatives (TN), false positives (FP), and false negatives (FN).

Balanced Accuracy gives equal weight to the classification performance of both classes under class-imbalanced settings:

$$\text{Balanced Accuracy}=\frac{1}{2}\left( \frac{\text{TP}}{\text{TP + FN}}+\frac{\text{TN}}{\text{TN + FP}} \right) \text{(S1)}$$

The first term in Eq. S1 corresponds to sensitivity, whereas the second term corresponds to specificity.

Precision quantifies the proportion of correctly predicted positive samples among all samples predicted as positive:

$$\text{Precision}=\frac{\text{TP}}{\text{TP + FP}} \text{(S2)}$$

Recall quantifies the proportion of actual positive samples that are correctly identified by the model:

$$\text{Recall}=\frac{\text{TP}}{\text{TP + FN}} \text{(S3)}$$

F1 Score is the harmonic mean of Precision in Eq. S2 and Recall in Eq. S3, and provides a balanced summary of the two measures:

$$\text{F1 Score}=2\times\frac{\text{Precision}\times\text{Recall}}{\text{Precision}+\text{Recall}} \text{(S4)}$$

AUROC is defined as the area under the ROC curve, which characterizes the trade-off between the True Positive Rate (TPR) and the False Positive Rate (FPR) across different threshold values:

$$\text{AUROC}=\int_{0}^{1} \text{TPR}\left( t \right) d\left( \text{FPR}\left( t \right) \right) \text{(S5)}$$

In Eq. S5, $\text{TPR}=\frac{\text{TP}}{\text{TP + FN}}$ and $\text{FPR}=\frac{\text{FP}}{\text{FP + TN}}$. An AUROC value closer to 1 indicates stronger classification performance.

AUPR is defined as the area under the precision-recall curve, which represents the trade-off between Precision and Recall across different thresholds:

$$\text{AUPR}=\int_{0}^{1} \text{Precision}\left( r \right) dr \text{(S6)}$$

In Eq. S6, $r$ denotes the Recall value. AUPR is particularly useful for evaluating positive-class prediction performance in heavily imbalanced datasets, as it more faithfully reflects model behavior when positive samples are scarce. Values closer to 1 indicate better performance.

Average Drop% measures the mean decrease in the model confidence for class $c$ when only the region highlighted by the explanatory map is retained as input:

$$\text{Average Drop\%}=\frac{1}{N}\sum_{i=1}^{N} \frac{\max\left( 0,Y_{i}^{c}-O_{i}^{c} \right)}{Y_{i}^{c}}\times100 \text{(S7)}$$

In Eq. S7, $N$ denotes the number of samples, and $Y_{i}^{c}$ is the original confidence score for class $c$ on sample $i$. The term $O_{i}^{c}$ denotes the confidence score for the same class when only the region emphasized by the explanatory map is preserved and the remaining regions are masked out. A lower Average Drop% indicates that the explanatory map more effectively captures the discriminative regions used by the model.

% Increase in Confidence measures the proportion of samples for which the confidence score for class $c$ increases under the same masking setting:

$$\text{\%Increase in Confidence}=\frac{1}{N}\sum_{i=1}^{N} \mathbb{I}\left( Y_{i}^{c}<O_{i}^{c} \right)\times100 \text{(S8)}$$

In Eq. S8, $\mathbb{I}\left( \cdot\right)$ denotes an indicator function that returns 1 when the condition is satisfied and 0 otherwise. A higher % Increase in Confidence indicates that the explanatory map more effectively identifies informative regions while excluding less relevant areas.

Positive Perturbation AUC quantifies the area under the confidence-change curve for class $c$ when pixels with high importance scores in the explanatory map $M$ are sequentially removed:

$$\text{Positive Perturbation AUC}=\int_{0.1}^{0.9} f_{c}\left( \Phi_{\text{high}}\left( I,M,k \right) \right) dk \text{(S9)}$$

In Eq. S9, $k$ denotes the proportion of removed pixels ($k\in\left[ 0.1,0.9 \right]$), and $\Phi_{\text{high}}\left( I,M,k \right)$ represents the image obtained by removing the top-ranked important pixels according to the explanatory map $M$ from the original image $I$ by a ratio of $k$. In addition, $f_{c}\left( \cdot\right)$ denotes the model confidence function for class $c$. A lower Positive Perturbation AUC indicates that the explanatory map more accurately identifies the regions that are most critical to the model decision.

Negative Perturbation AUC quantifies the area under the confidence-change curve for class $c$ when pixels with low importance scores in the explanatory map $M$ are sequentially removed:

$$\text{Negative Perturbation AUC}=\int_{0.1}^{0.9} f_{c}\left( \Phi_{\text{low}}\left( I,M,k \right) \right) dk \text{(S10)}$$

In Eq. S10, $\Phi_{\text{low}}\left( I,M,k \right)$ denotes the image obtained by removing the lowest-importance pixels in the explanatory map $M$ by a ratio of $k$. A higher Negative Perturbation AUC indicates that the explanatory map more effectively isolates non-essential background or noise regions while preserving the core evidence used by the model.

# S2. Experiment Results

## S2.1. Data Analysis

Fig. S1. Visualize the feature distribution of the strawberry disease type classes. (a) Tipburn, (b) Phytophthora blight, (c) Fusarium wilt, (i) Original t-SNE, (ii) Original UMAP, (iii) Overexposure t-SNE, Overexposure UMAP.

**Data visualization analysis**: Figure S1 shows the two-dimensional embedding results of feature vectors for each strawberry disease type. Across all disease types, both t-SNE and UMAP show that the Normal and Abnormal classes are generally separated, although partial overlap is still observed between the two classes. In the t-SNE visualizations, the samples tend to form relatively broad clusters, with noticeable mixing near the class boundaries. In contrast, UMAP produces more compact class-wise groupings, although several samples remain intermixed between the clusters. Similar overall distribution patterns are observed under both the original and overexposure conditions, suggesting that the global feature structure is largely preserved even under conditions designed to simulate bright-region saturation caused by direct light, reflected light, or strong illumination in real cultivation environments. However, for phytophthora blight and fusarium wilt, the relative positions of the Normal and Abnormal clusters change between the original and overexposure conditions, suggesting that overexposure can influence the organization of the feature space.

Table S1. Statistical Analysis.

| Dataset | Condition | Feature | Normal ($\mu\pm\sigma$) | Abnormal ($\mu\pm\sigma$) | $t$-statistic | $p$-value |
| --- | --- | --- | --- | --- | --- | --- |
| Tipburn | Original | L_mean | $126.732\pm15.686$ | $137.824\pm14.360$ | $-11.662$ | $<0.001$ |
|  |  | a_mean | $117.651\pm3.935$ | $120.591\pm2.476$ | $-14.139$ | $<0.001$ |
|  |  | b_mean | $141.164\pm6.448$ | $136.865\pm3.315$ | $13.255$ | $<0.001$ |
|  |  | Area | $487,982.427\pm308,571.412$ | $764,579.765\pm400,016.071$ | $-12.242$ | $<0.001$ |
|  |  | Circularity | $0.087\pm0.094$ | $0.056\pm0.049$ | $6.602$ | $<0.001$ |
|  |  | Aspect_ratio | $0.568\pm0.205$ | $0.626\pm0.177$ | $-4.817$ | $<0.001$ |
|  | Overexposure | L_mean | $201.631\pm12.782$ | $211.347\pm10.595$ | $-13.086$ | $<0.001$ |
|  |  | a_mean | $117.746\pm3.600$ | $121.092\pm2.177$ | $-17.780$ | $<0.001$ |
|  |  | b_mean | $140.124\pm5.501$ | $136.620\pm2.723$ | $12.767$ | $<0.001$ |
|  |  | Area | $702,945.248\pm365,676.839$ | $1,103,304.766\pm558,874.255$ | $-13.404$ | $<0.001$ |
|  |  | Circularity | $0.066\pm0.077$ | $0.047\pm0.037$ | $4.965$ | $<0.001$ |
|  |  | Aspect_ratio | $0.603\pm0.185$ | $0.664\pm0.159$ | $-5.615$ | $<0.001$ |
|  | Original | L_mean | $126.870\pm15.466$ | $101.608\pm22.931$ | $20.422$ | $<0.001$ |
|  |  | a_mean | $117.951\pm4.066$ | $121.187\pm3.217$ | $-13.956$ | $<0.001$ |
|  |  | b_mean | $141.359\pm6.196$ | $137.255\pm3.867$ | $12.563$ | $<0.001$ |
|  |  | Area | $497,033.912\pm305,720.164$ | $326,607.242\pm242,693.997$ | $9.763$ | $<0.001$ |
|  |  | Circularity | $0.094\pm0.111$ | $0.042\pm0.042$ | $9.849$ | $<0.001$ |
|  |  | Aspect_ratio | $0.549\pm0.205$ | $0.621\pm0.178$ | $-5.946$ | $<0.001$ |
|  | Overexposure | L_mean | $201.707\pm12.893$ | $178.944\pm16.019$ | $24.753$ | $<0.001$ |
|  |  | a_mean | $118.101\pm3.682$ | $121.782\pm2.724$ | $-17.971$ | $<0.001$ |
|  |  | b_mean | $140.225\pm5.231$ | $136.276\pm3.369$ | $14.194$ | $<0.001$ |
|  |  | Area | $699,769.188\pm340,353.732$ | $461,843.284\pm350,752.825$ | $10.885$ | $<0.001$ |
|  |  | Circularity | $0.073\pm0.093$ | $0.043\pm0.041$ | $6.610$ | $<0.001$ |
|  |  | Aspect_ratio | $0.604\pm0.189$ | $0.665\pm0.166$ | $-5.467$ | $<0.001$ |
|  | Original | L_mean | $126.613\pm15.777$ | $107.281\pm19.432$ | $17.270$ | $<0.001$ |
|  |  | a_mean | $117.659\pm3.770$ | $124.137\pm4.016$ | $-26.295$ | $<0.001$ |
|  |  | b_mean | $141.380\pm5.875$ | $134.477\pm4.697$ | $20.521$ | $<0.001$ |
|  |  | Area | $468,908.210\pm291,263.882$ | $283,561.888\pm278,654.836$ | $10.282$ | $<0.001$ |
|  |  | Circularity | $0.092\pm0.095$ | $0.048\pm0.057$ | $8.955$ | $<0.001$ |
|  |  | Aspect_ratio | $0.549\pm0.202$ | $0.615\pm0.176$ | $-5.462$ | $<0.001$ |
|  | Overexposure | L_mean | $201.806\pm12.903$ | $184.431\pm14.813$ | $19.777$ | $<0.001$ |
|  |  | a_mean | $117.729\pm3.431$ | $124.411\pm3.457$ | $-30.680$ | $<0.001$ |
|  |  | b_mean | $140.449\pm5.041$ | $133.344\pm4.161$ | $24.308$ | $<0.001$ |
|  |  | Area | $685,060.852\pm376,749.252$ | $473,350.888\pm404,551.673$ | $8.563$ | $<0.001$ |
|  |  | Circularity | $0.065\pm0.071$ | $0.032\pm0.038$ | $9.327$ | $<0.001$ |
|  |  | Aspect_ratio | $0.599\pm0.187$ | $0.658\pm0.150$ | $-5.542$ | $<0.001$ |

**Statistical analysis**: Table S1 presents the results of the t-test analysis of color and morphological differences between Normal and Abnormal samples for each strawberry disease type. Statistically significant differences in color and morphological features between Normal and Abnormal samples were observed across Tipburn, Phytophthora blight, and Fusarium wilt under both original and overexposure conditions. These results indicate that color and morphological features contain meaningful information for distinguishing normal and abnormal states across strawberry disease types. However, such statistically significant differences do not always lead to clear class separation in the feature space, as partial overlap was still observed in the visualization analysis. This suggests the need for an additional feature enhancement mechanism to learn more discriminative feature representations.

Fig. S2. Violin plots showing the distribution of mean pixel intensity for Normal and Abnormal samples. (a) Tipburn, (b) Phytophthora blight, (c) Fusarium wilt, (i) Original (ii) Overexposure.

Figure S2 presents violin plots of the mean pixel intensity distributions of Normal and Abnormal samples for each disease type under the original and overexposure conditions. Statistically significant differences between the two distributions were observed across all disease types. However, substantial overlap remained between the distributions, indicating that mean pixel intensity alone is insufficient for clear class discrimination. This supports the need for a multidimensional analysis that jointly considers color and morphological features.
